# Normal and Pathological NRF2 Signalling in the Central Nervous System

**DOI:** 10.3390/antiox11081426

**Published:** 2022-07-22

**Authors:** Tony Heurtaux, David S. Bouvier, Alexandre Benani, Sergio Helgueta Romero, Katrin B. M. Frauenknecht, Michel Mittelbronn, Lasse Sinkkonen

**Affiliations:** 1 Department of Life Sciences and Medicine (DLSM), University of Luxembourg, 4367 Belvaux, Luxembourg; sergio.helgueta@uni.lu (S.H.R.); michel.mittelbronn@lns.etat.lu (M.M.); lasse.sinkkonen@uni.lu (L.S.); 2 Luxembourg Center of Neuropathology (LCNP), 3555 Dudelange, Luxembourg; david.bouvier@lns.etat.lu (D.S.B.); katrin.frauenknecht@lns.etat.lu (K.B.M.F.); 3 National Center of Pathology (NCP), Laboratoire National de Santé (LNS), 3555 Dudelange, Luxembourg; 4 Luxembourg Centre of Systems Biomedicine (LCSB), University of Luxembourg, 4367 Belvaux, Luxembourg; 5 Centre des Sciences du Goût et de l’Alimentation, AgroSup Dijon, CNRS, INRAE, Université Bourgogne Franche-Comté, 21000 Dijon, France; alexandre.benani@u-bourgogne.fr; 6 Luxembourg Institute of Health (LIH), 1526 Luxembourg, Luxembourg

**Keywords:** NRF2, reactive oxygen species, glial cells, diet, ageing, cancer, neurodegeneration, epigenetic regulation

## Abstract

The nuclear factor erythroid 2-related factor 2 (NRF2) was originally described as a master regulator of antioxidant cellular response, but in the time since, numerous important biological functions linked to cell survival, cellular detoxification, metabolism, autophagy, proteostasis, inflammation, immunity, and differentiation have been attributed to this pleiotropic transcription factor that regulates hundreds of genes. After 40 years of in-depth research and key discoveries, NRF2 is now at the center of a vast regulatory network, revealing NRF2 signalling as increasingly complex. It is widely recognized that reactive oxygen species (ROS) play a key role in human physiological and pathological processes such as ageing, obesity, diabetes, cancer, and neurodegenerative diseases. The high oxygen consumption associated with high levels of free iron and oxidizable unsaturated lipids make the brain particularly vulnerable to oxidative stress. A good stability of NRF2 activity is thus crucial to maintain the redox balance and therefore brain homeostasis. In this review, we have gathered recent data about the contribution of the NRF2 pathway in the healthy brain as well as during metabolic diseases, cancer, ageing, and ageing-related neurodegenerative diseases. We also discuss promising therapeutic strategies and the need for better understanding of cell-type-specific functions of NRF2 in these different fields.

## 1. Beneficial and Harmful Roles of ROS

The nuclear factor erythroid 2-related factor 2 (NRF2) transcription factor pathway is able to neutralize reactive oxygen species (ROS) in order to maintain the cellular redox balance. Before going into details of the NRF2 regulatory pathway, we address the beneficial but also deleterious effects of ROS in this first section. It seemed important to us to summarize some background information, such as: how are ROS produced? What are their physiological roles? What are their deleterious effects? How is oxidative stress generated?

### 1.1. Radicals and Other Reactive Species at a Glance

Molecular oxygen (O_2_) is fundamental for survival of all aerobic organisms. According to its electronic structure, molecular oxygen has two unpaired electrons, allowing for capturing two electrons, albeit only one at a time. This electronic reduction of O_2_ then leads to the formation of metabolites, called reactive oxygen species (ROS), classified in radicals, including superoxide anion (O_2_^●−^), hydroxyl (HO^●^), alkoxyl (RO^●^), peroxyl (ROO^●^), lipid (L^●^), lipid alkoxyl (LO^●^), and lipid peroxyl (LOO^●^), and nonradical species such as hydrogen peroxide (H_2_O_2_), hypochlorous acid (HOCl), ozone (O_3_), and lipid peroxide (LOOH).

**Figure 1:**
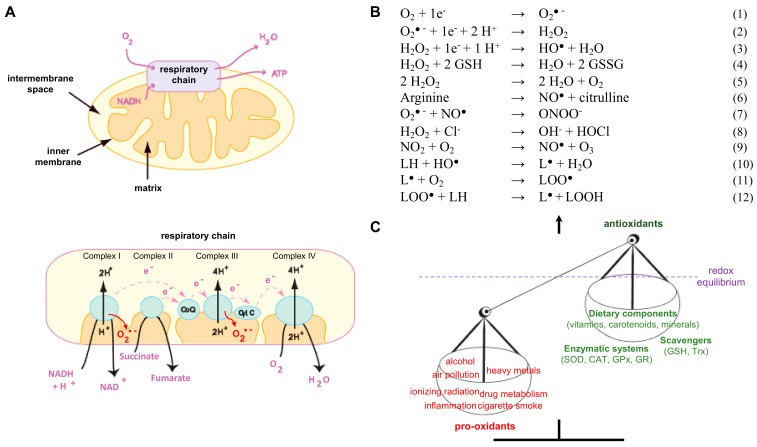
Reactive species production and disruption of the redox homeostasis. (**A**) Generation of superoxide anions (O_2_^●^^−^) by the complexes I and III of the mitochondrial respiratory chain. (**B**) ROS are byproducts of the normal metabolism of oxygen. (1) ROS formation follows the monoelectronic reduction of O_2_. (2) Superoxide dismutases catalyse the dismutation of superoxide into hydrogen peroxide H_2_O_2_. (3) Hydroxyl radical HO^●^ is produced by decomposition of H_2_O_2_ via the Fenton reaction (Fe(II)-dependent reaction). (4) Glutathione peroxidase catalyses the reduction of hydrogen peroxide to water via oxidation of reduced glutathione (GSH) into its disulphide form (GSSG). (5) Catalase reacts with the hydrogen peroxide to catalyse the formation of water and O_2_. (6) The production of nitric oxide (NO^●^), a reactive nitrogen species, is carried out from L-arginine by nitric oxide synthases. (7) Peroxynitrite ONOO^−^ is produced by the reaction of the free radical superoxide O_2_^●−^ with the free radical nitric oxide NO^●^. (8) Hypochlorous acid (HOCl) is produced through myeloperoxidase (MPO)-catalysed peroxidation of chloride anions using H_2_O_2_. (9) Nitrogen dioxide (NO_2_) reacts with molecular oxygen to form, under the action of UV radiation and heat, nitric oxide (NO^●^) and ozone (O_3_). Lipid peroxidation (10)–(12) is a chain of reactions of oxidative degradation of lipids: (10) unsaturated lipid (LH) reacts with prooxidants (hydroxyl radical, HO^●^), leading to the formation of lipid radical (L^●^) and water; (11) lipid radical then reacts with oxygen to form a lipid peroxy radical (LOO^•^), which abstracts one hydrogen from another lipid molecule, generating a new lipid radical (L^●^) and lipid hydroperoxide (LOOH) (12). (**C**) The redox balance is an equilibrium between ROS production, due to pro-oxidant conditions, and antioxidant defences. A disruption of this redox balance in favour of oxidative conditions will promote an oxidative stress. CAT, catalase; CoQ, coenzyme Q; Cyt C, cytochrome C; GPx, glutathione peroxidase; GR, glutathione reductase; GSH, reduced glutathione; GSSG, oxidized glutathione; SOD, superoxide dismutases; Trx, thioredoxin.

Reactive oxygen species are very short-lived but reactive molecules. They are mainly produced at membranes of mitochondria and the endoplasmic reticulum, in peroxisomes as well as in the cytosol [1,2]. The mitochondrial respiratory chain is considered the main source of physiological ROS production. During respiration, around 1 to 2% of O_2_ is not entirely reduced to water, therefore leading to the production of superoxide anion O_2_^●−^ [2,3] (Figure 1A). Furthermore, an alteration of the inner mitochondrial membrane potential and electron transport rate can lead to an increase in the production of superoxide anions. ROS can also be produced in the cytosol by a large panel of active oxidoreductases such as NADPH oxidases (NOX), cytochrome P450 (CYP) oxidase, cyclooxygenases (COX), lipoxygenases (LOX), and monoamine oxidases (MAO) [4,5]. The lifespan of such reactive species is rather short, as they are quickly transformed by antioxidant proteins (e.g., superoxide dismutases (SOD), glutathione peroxidases (GPX), catalase (CAT)) into H_2_O_2_ and finally into water (Figure 1B); however, intermediate species can also be produced. Fenton and Haber–Weiss reactions, in which ferrous iron (Fe^2+^) reacts with H_2_O_2_, cause the formation of HO^●^, constituting one of the most reactive species. HO^●^ and hydroperoxyl (HOO^●^) radicals play an important role in the lipid peroxidation process by attacking lipids containing carbon–carbon double bonds such as polyunsaturated fatty acids [6,7] and in the subsequent production of reactive unsaturated aldehydes (e.g., 4-hydroxynonenal (4-HNE) and malondialdehyde (MDA)) [6].

Like ROS, reactive nitrogen species (RNS) are divided into radical and nonradical species, including nitric oxide (NO^●^), nitrogen dioxide (NO_2_^●^), peroxynitrite (ONOO^−^), and nitrous acid (HNO_2_). Nitric oxide is produced by the activity of nitric oxide synthases (NOS), which convert the amino acid L-arginine into L-citrulline and NO^●^ (Figure 1B). A powerful oxidant, named peroxynitrite, is the result of the reaction between superoxide anion and nitric oxide [8]. Finally, the production of reactive species can be linked not only to metabolic activities but also to environmental factors, such as pollutants (pesticides, heavy metals), UV radiation, and lifestyle behaviours (tobacco smoke, excess alcohol, food habits, unbalanced exercise) [9,10,11,12].

### 1.2. Significance of ROS

Depending on their levels, ROS can be either beneficial or harmful to living systems. Over the past thirty years, many studies highlighted that ROS, at low concentrations, are important mediators of numerous signalling pathways. In particular, hydrogen peroxide, the most stable ROS with a relatively long biological lifespan (cellular half-life of 10^−3^ s; 1000 times more than other ROS), has the ability to diffuse through membranes, thus acting as an autocrine and paracrine signal [13,14]. Reactive species play useful biological roles. Indeed, ROS have been shown to be implicated in numerous physiological functions in embryonic and foetal development [15,16], neuronal development and function [17], and cellular proliferation and differentiation [18] due to notably supporting stem cell renewal and differentiation [19,20]. Roles in immune responses [21], activation of cell survival signalling pathways [22], normal growth and metabolism [23], tissue regeneration [24], blood pressure control [25,26,27], ageing prevention [28], and the execution of cell death programs such as apoptosis [22,29] can also be added to the long list of physiological functions attributed to ROS. Closely related to the synthesis of key signalling molecules, called eicosanoids (leukotrienes, lipoxins, prostaglandins, thromboxanes), lipid (hydro)peroxides are also considered as actors in normal physiological processes [6,30,31]. This eicosanoid synthesis is linked to COX and LOX activities. Importantly, these enzymes require [31,32] low levels of pre-existing lipid hydroperoxides (LOOH) to “prime” their catalytic cycles. Eicosanoids are lipid-signalling molecules deriving from arachidonic acid. These lipid mediators are key regulators of a wide variety of physiological responses as well as pathological processes. Many important cellular actions (e.g., proliferation, metabolism, and migration) are controlled by eicosanoids in the whole body [33,34,35]. An imbalance of this major lipid-signalling pathway contribute to disease progression [36]. It should also be noted that in the absence of ROS, alteration of the previously described functions will lead to decreased cell growth, metabolism, and proliferation as well as defective host defences [23].

Upon increased ROS levels, mammalian cells, equipped with a complex antioxidant defence system, will first try to maintain the redox balance [4,37,38]. For this purpose, enzymatic antioxidants (e.g., SOD, CAT, GPX), non-enzymatic scavengers (e.g., glutathione (GSH), thioredoxin, ascorbic acid (vitamin C), α-tocopherol (vitamin E), carotenoids), and also enzymes that repair cell damage (e.g., polymerases, nucleases, proteases) have a fundamental role in cellular protection. Redox balance, also called redox homeostasis, is an equilibrium between the production of ROS and these different detoxification systems (Figure 1C). However, an excessive and permanent increase in ROS production will induce an oxidative stress, which is classically defined as the alteration of the intracellular redox balance in favour of oxidative conditions. When ROS generation exceeds cellular capacity for detoxification, oxidative damage to nucleic acids (strand breaks, base oxidation), proteins (oxidation, nitration, carbonylation), lipids (peroxidation), membranes, and organelles (altered structures and properties) can occur, leading to cell dysfunction and cell death. Ageing and many human diseases such as cancer, diabetes, neurodegenerative disorders, cardiovascular diseases, and inflammation-related diseases have been linked to this progressive physiological dysfunction.

### 1.3. Redox Homeostasis in Ageing

According to the World Health Organization (WHO), the worldwide population over 60 years old will almost double, reaching 22% of the total population, by 2050. The proportion of people 80 years old or older is expected to triple between 2020 and 2050.

Ageing is a decline in physiological functions leading to the progressive loss of function in tissues and organs over time [39,40]. In 1956, the chemist Denham Harman published the “Free Radicals Theory of Ageing” [41]. He postulated that free radicals play a role in the ageing process. Although this hypothesis has been questioned many times, the fact that healthy ageing may be linked to oxidative stress resistance appears to still be relevant. The exact mechanism of oxidative-stress-induced ageing is still not clearly defined. However, it has been well established and accepted by the scientific community that a disrupted antioxidant/oxidant equilibrium coupled with an accumulation of oxidative damage to cellular constituents (DNA, proteins, lipids) and a chronic inflammation are pervasive features of ageing [42,43]. Key inflammatory players are indeed involved in the age-related process: activation of the pro-inflammatory nuclear factor kappa-light-chain-enhancer of activated B cells (NF-κB) pathway, upregulation of cytokines (interleukin 1 beta (IL-1β), IL-6, tumour necrosis factor alpha (TNF-α)), C-C motif chemokine ligands (CCL2, CCL20) and their receptors, and other proinflammatory factors (matrix metalloproteinases, COX2, and NOS2) [42,44,45,46,47,48]. All these pro-inflammatory actors are indeed more expressed in aged than in young tissues [44,49]. The antioxidant/oxidant equilibrium is then disrupted during ageing, mainly due to a decrease in GSH synthesis as well as a downregulation of antioxidant enzymes [50,51]. A persistent oxidative stress associated with chronic inflammation may trigger ageing processes but also lead to age-related chronic diseases.

## 2. The NRF2-KEAP1 (Kelch-Like ECH-Associated Protein 1)-ARE (Antioxidant Response Element) Signalling Pathway

### 2.1. Mode of Regulation

As discussed in the previous section, the control of ROS levels is crucial to maintain cellular homeostasis. NRF2 is a transcription factor that emerged as a master regulator of the cellular antioxidant response. 

**Figure 2:**
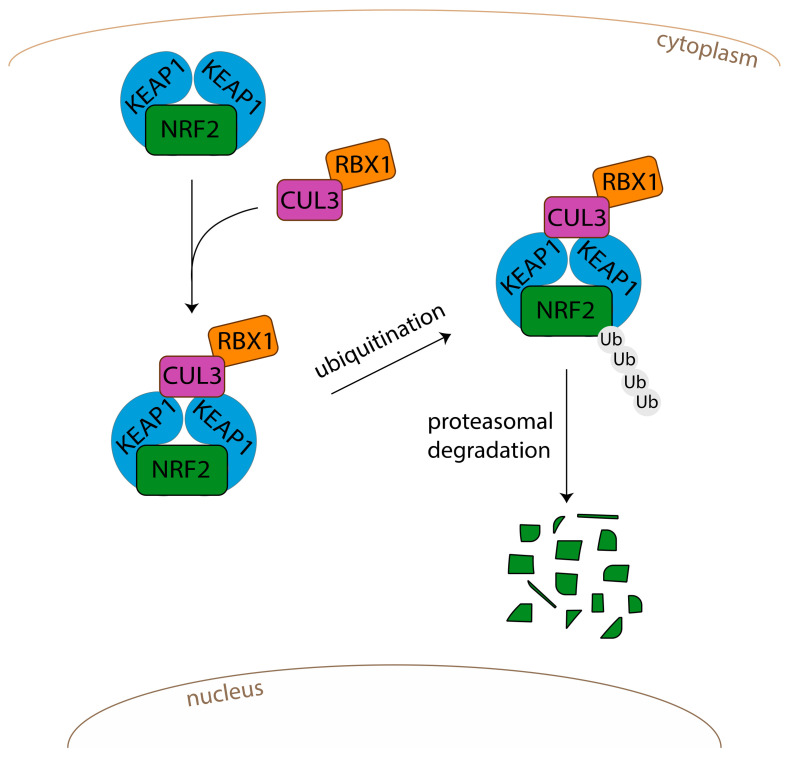
**NRF2-KEAP1 signalling pathway in basal conditions**. Under homeostatic conditions, NRF2 is sequestered by cytoplasmic KEAP1/CUL3-RBX1 E3 ubiquitin ligase complex and targeted to proteasomal degradation. CUL3, Cullin-3; KEAP1, Kelch-like ECH-associated protein 1; NRF2, nuclear factor erythroid 2-related factor 2; RBX1, RING Box Protein 1; Ub, Ubiquitin.

Encoded by the *NFE2L2* gene, NRF2 is a CNC-bZIP (Cap’n’Collar basic-region leucine zipper) transcription factor ubiquitously expressed in the body. Under normal conditions, NRF2 is kept in the cytoplasm by the KEAP1/CUL3-RBX1 E3 ubiquitin ligase complex, which is known to be the major repressor of NRF2 [52] (Figure 2). Beyond KEAP1, other regulators also exist. Indeed, BTB and CNC homology transcription factors, called BACH1 and BACH2, function as NRF2 repressors by competing with NRF2 binding to the ARE sequences [53,54,55,56]. BACH1 has been described to be widely expressed in mammalian tissues [54], whereas BACH2 is predominantly expressed in B and T lymphocytes, T cells, macrophages, and neural cells [57,58,59].

**Figure 3:**
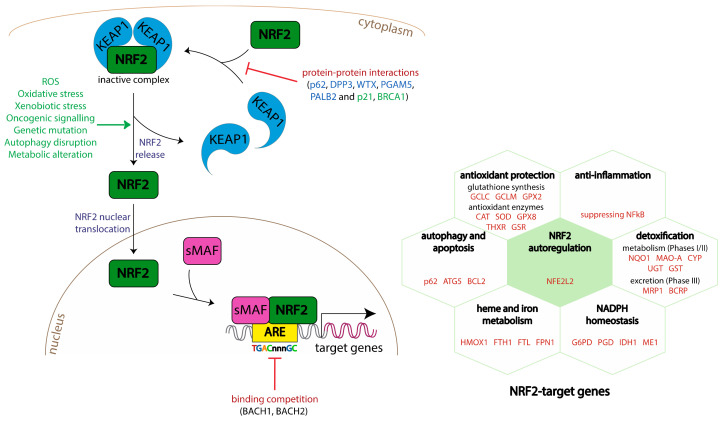
**Activation and regulation of the NRF2 signalling pathway**. Under stress conditions, NRF2 is released from KEAP1 and translocates to the nucleus, where it interacts with cofactors and binds specific response elements (ARE) to regulate the transcription of its many target genes. ARE, Antioxidant Response Element; ATG5, Autophagy-related 5; BACH1/2, BTB and CNC homology 1/2; BCL2, B-cell lymphoma 2; BCRP, Breast cancer resistance protein; BRCA1, Breast cancer type 1; CAT, Catalase; CYP, Cytochrome p450; DPP3, Dipeptidyl peptidase 3; FTH1, Ferritin Heavy Chain 1; FTL, Ferritin light chain; FPN1, Ferroportin1; G6PD, Glucose-6-phosphate dehydrogenase; GCLC, Glutamate-cysteine ligase catalytic subunit; GCLM, Glutamate-cysteine ligase regulatory subunit; GPX2/8, Glutathione peroxidase 2/8; GSR, glutathione reductase; GST, glutathione S-transferase; HMOX1, Heme Oxygenase 1; IDH1, isocitrate dehydrogenase 1; MAO-A, monoamine oxidase A; ME1, malic enzyme 1; MRP1, Multidrug resistance protein 1; NQO1, NAD(P)H:quinone oxidoreductase 1; NF-κB, Nuclear factor kappa-light-chain-enhancer of activated B cells; p21, cyclin-dependent kinase inhibitor 1; p62, sequestosome 1; PALB2, Partner and localizer of BRCA2; PGAM5, Phosphoglycerate mutase 5; PGD, 6-phosphogluconate dehydrogenase; ROS, Reactive oxygen species; sMAF, small musculoaponeurotic fibrosarcoma; SOD, Superoxide dismutase; THXR, Thioredoxin reductase; UGT, UDP-glucuronosyltransferase; WTX, Wilms tumour gene on the X chromosome.

Sequestered in the cytosol, NRF2 is inactive and rapidly subjected to ubiquitination and proteasomal degradation (Figure 2). NRF2 is a protein with high turn-over. Complexed with KEAP1, the NRF2 half-life (t_1/2_) is approximately 20 min [60]. Oxidative conditions, following exposure to reactive chemicals, chemopreventive molecules (electrophiles agents), or an oxidative stress, will lead to the modification of two cysteine residues (C273 and C288) localized in the intervening region of KEAP1. As a consequence, the KEAP1-NRF2 complex will be disrupted, and NRF2 will be released (Figure 3). Several works have confirmed the role of such cysteine residues: mutations on cysteine 273 or cysteine 288 inactivate KEAP1 and promote the release of NRF2 [61,62,63]. Once free, stabilized NRF2 translocates into the nucleus, heterodimerizes with small musculoaponeurotic fibrosarcoma (sMAF) proteins, and binds to specific DNA sequences called antioxidant or electrophile response elements (ARE/EpRE, 5′-TGACNNNGC-3′) located in the 5′-flanking regions of gene promoters (Figure 3).

Post-translational modifications may also regulate NRF2 activities independently of KEAP1 expression [64]. Thus, the mitogen-activated protein (MAP) kinases, extracellular signal-regulated protein kinase (ERK) and c-jun N-terminal kinase (JNK), but also serine/threonine kinase (Akt), phosphatidylinositol 3-kinase (PI3K), 5′-AMP-activated protein kinase (AMPK), and protein kinase C (PKC) are able to phosphorylate the threonine, tyrosine and serine residues of NRF2, enhancing its activity [65,66,67,68,69]. In contrast, the glycogen synthase kinase-3 beta (GSK-3β) functions as an NRF2 inhibitor [70,71]. KEAP1 can also be subject to modifications by phosphorylation. Huo and collaborators described that the epidermal growth factor receptor (EGFR) tyrosine kinase was able to activate the NRF2 signalling pathway by phosphorylating KEAP1, thus resulting in nuclear NRF2 stabilization [72]. 

### 2.2. Biological Functions

NRF2 positively regulates hundreds of genes containing ARE sequences in their promoter regions [73]. The key well-known NRF2 function is linked to the maintenance of the redox balance through the synthesis or the use of γ-glutamyl-cysteinyl-glycine tripeptide (GSH). Thus, NRF2 is implicated in the de novo synthesis of GSH by the increase in glutamate-cysteine ligase (GCL) expression, the transformation of oxidized glutathione (GSSG) in GSH (glutathione reductase, GR), the expression of several enzymes using GSH for reducing peroxides (GPXs; peroxiredoxins), the breakdown of peroxides (CAT), and the import of cysteine (xCT transporter) important for GSH production (Figure 3).

NRF2 is also involved in the upregulation of the expression of drug/xenobiotic-metabolizing enzymes. Phase I and II drug-metabolizing enzymes play important detoxification roles by transforming the “parent drugs” into metabolites, which are less lipophilic and easier to eliminate through urine. NRF2 also modulates the transcription of Phase III drug-metabolizing enzymes implicated in the excretion of drugs/xenobiotics and metabolites from the cell. This will lead to a decrease in the therapeutic efficacy of drugs through their metabolism as well as through their elimination.

Numerous papers have also described the role of NRF2 in the reprogramming of cellular metabolism to support antioxidant responses [74,75]. Indeed, ARE sequences have been identified in genes implicated in glucose metabolism (glycolysis, pentose phosphate pathway, nucleotide biosynthesis pathway), lipid metabolism (lipid export, import, and synthesis), and heme and iron metabolism due to degrading free heme into biliverdin, CO, and Fe^2+^ (heme oxygenase 1, *HMOX1*), storing iron in its oxidized state (ferritin heavy chain 1, *FTH1*; ferritin light chain, *FTL*), and exporting unstable iron (ferroportin 1, *FPN1*) [53,76].

Autophagy processes and proteasome assembly are also governed by NRF2 transcription factor [77,78]. Autophagy plays an important role in removing misfolded or aggregated proteins as well as clearing damaged organelles (endoplasmic reticulum, mitochondria, and peroxisomes). Under normal conditions, the autophagy adaptor protein p62 binds to ubiquitylated protein aggregates in the cytoplasm before delivering them to the autophagosomes for degradation. When autophagy is disrupted, p62 accumulates in the cytoplasm and directly interacts with the NRF2-binding site on KEAP1. In this condition, the p62-KEAP1 complex is retained in the phagosomes, resulting in NRF2 stabilization and subsequently in transcriptional activation of NRF2 target genes [79,80]. Moreover, it has been described that ARE sequences are located on p62 promoter [53,81], indicating that NRF2 can also upregulate its transcriptional expression. In this way, p62 positively regulates NRF2 function independently of the cellular oxidative status. In contrast, a knockdown of p62 will decrease KEAP1 degradation and therefore NRF2 activation [82,83]. 

NRF2 levels vary significantly depending on physiological and pathological context. In recent years, many methods have been developed to monitor these changes in NRF2 pathway activity (Table 1), the aim being to assess NRF2 involvement in the pathogenesis of human diseases, to track disease progression but also to improve preclinical identification of targets, chemicals, and drugs.

## 3. Oxidative Stress, Inflammation, and NRF2 in the Brain

### 3.1. The Brain, an Ideal Target for Oxidative Attacks

The brain is particularly vulnerable to oxidative stress. To support intensive neuronal activity, the brain consumes almost 20% of the total O_2_ intake. This high O_2_ consumption associated with a high content of free metals (e.g., iron, copper, zinc, manganese), oxidizable polyunsaturated fatty acids, and auto-oxidation of neurotransmitters (dopamine, norepinephrine, and serotonin) may increase the risk of producing excessive ROS and, as a result, will make the brain vulnerable to ROS-mediated injury [102,103]. Compared to other organs (liver, kidney), the brain has fewer antioxidant defences, with lower CAT, GPX, and SOD activities. As an example, CAT concentration in the brain is 50 times lower than in the liver [104]. Disparities also exist within the brain itself. Unlike neurons, astrocytes and microglia appear to contain high GSH levels [105]. Neuronal GSH level is at least 50% lower than in other cells [103]. The major antioxidant scavenger, GSH, which is a γ-glutamyl-cysteinyl-glycine tripeptide, is important for cellular defence against reactive oxygen/nitrogen species. In the presence of reactive species, two molecules of GSH are oxidized to one molecule of GSSG, which is afterwards reduced to GSH by glutathione reductase. Astrocytes can convert glutamate released by neurons into glutamine and γ-glutamyl-cysteine, two GSH precursors. Since neuronal GSH levels are modest, astrocytes provide precursors (glutamine, cysteinyl-glycine dipeptide) to neurons to produce GSH themselves [106] to efficiently degrade H_2_O_2_. In addition to GSH release, astrocytes have also been described to produce other antioxidant molecules (e.g., ascorbate and vitamin E) and to activate ROS-detoxifying enzymes to protect neurons [105]. During oxidative insults, both astrocytes and microglia focus on maintaining the brain homeostasis in order to protect neurons. However, a huge production of reactive species as well as a GSH deficiency will contribute to a severe oxidative stress and will then play a role in the onset/aggravation of related pathological diseases [107,108].

### 3.2. NRF2, Driver for a Healthy Brain?

Concerning the brain, numerous studies have described NRF2 as a transcription factor mainly expressed in glial cells [109,110,111,112]. Considering the Brain RNAseq data from Barres’ Lab (www.brainrnaseq.org accessed on 20 June 2022), *NFE2L2* expression level is indeed stronger in glial cells (microglia, astrocytes) compared to neuronal cells in both mouse and human. KEAP1, the major repressor of NRF2, appears to be expressed in all cells at a substantially identical level.

Over time, the relationship between NRF2 and brain health has been clearly established. The transcription factor has significant neuroprotective effects in brain diseases and injuries. As evidence, several studies have suggested that NRF2-deficient animals are more prone to inflammatory, cytotoxic, genotoxic, and neurotoxic effects due to endogenous and exogenous stressors [113,114,115,116,117]. Johnson and collaborators demonstrated that the absence of NRF2 in an experimental autoimmune encephalomyelitis model exacerbates the development of the disease, resulting in a more severe clinical course, a faster onset, and a higher percentage of diseased mice [118]. An NRF2 deficiency also worsened inflammatory parameters in a mouse model with combined tauopathy and amyloidopathy [119], increased Alzheimer’s disease (AD)-mediated cognitive decline [120], and significantly attenuated the self-renewal of glioma stem cells [121]. Furthermore, NRF2 deficiency mimics the ageing phenotype by exacerbating neuroinflammation, obesity-induced oxidative stress, blood-brain-barrier disruption, and cognitive decline in mice [122]. These observations coming from animal experiments have been confirmed in recent human studies [123,124,125,126,127,128].

### 3.3. Anti-Inflammatory Contribution of NRF2

Inflammation is a complex process whose final goal is to repair tissues by reducing and eliminating the damage caused. Age, genetics, lifestyle (e.g., smoking and diet), and environmental pollutants are factors that can promote acute or chronic inflammation [129]. While acute inflammation is time-limited and beneficial to the host, chronic inflammation is described as a slow, silent, and long-term process increasing health threat to the host. Chronic inflammation is a common feature of many pathological conditions such as asthma, allergy, diabetes, inflammatory bowel diseases (ulcerative colitis and Crohn’s disease), cancer, obesity, rheumatoid arthritis, and neurodegenerative diseases.

The NF-κB transcription factor regulates numerous genes involved in different processes of the immune and inflammatory responses in the whole body [130]. A dysregulated NF-κB pathway is thus considered as a hallmark of chronic inflammatory diseases. Classically, the NF-κB complex is a homo- or heterodimer composed of subunits such as p65 (also known as RelA), RelB, c-Rel, p100 (p52), and p105 (p50) [131]. In basal conditions, inactive NF-κB is associated with the inhibitory protein IκBα and sequestered in the cytoplasm. Under inflammatory/oxidative conditions, IκBα is phosphorylated by an IκBα kinase (IKK), leading to the dissociation of the NF-κB/IκBα complex and to the translocation of NF-κB into the nucleus, where it binds to specific response elements and upregulates the transcription process. The activated NF-κB cascade notably promotes the secretion of pro-inflammatory mediators (e.g., cytokines, chemokines), which will then recruit immune cells, resulting in the production of ROS and RNS.

A functional NRF2 system is important to regulate both neuroinflammation, i.e., activation of microglia and astrocytes, and oxidative stress in the brain. NRF2 and NF-κB transcription factors regulate cellular responses to inflammation and oxidative stress in order to maintain brain homeostasis [123,132,133]. Both pathways have been described to inhibit each other [134]. The anti-inflammatory role of NRF2 is now well established [135,136,137]. NRF2 inhibits the activation of NF-κB pathway by increasing antioxidant defences as well as HO-1 expression. The primary role of HO-1 is to reduce the pro-oxidant heme levels. Furthermore, the byproducts of HO-1 activity, carbon monoxide (CO, anti-apoptotic), ferrous iron (Fe^2+^), and bilirubin (anti-inflammatory), mitigate deleterious effects of oxidative stress by neutralizing ROS and detoxifying toxic chemicals [138,139,140,141]. The NRF2 pathway has also been described to inhibit NF-κB signalling through a KEAP1-induced IKK proteasomal degradation mechanism [133,142,143]. When NF-κB and the NRF2 pathways are simultaneously activated, NF-κB-p65 subunit may repress the transcriptional activity of NRF2. Recent works indeed have detailed that NF-κB can interact with KEAP1 in the cytoplasm. The formation of this complex promotes the entry of KEAP1 in the nucleus and facilitates the dissociation of NRF2 from the ARE sequences. The KEAP1-NRF2 complex thus formed will be exported from the nucleus and will then be degraded [144,145]. In response to oxidative stress, the NF-κB can also silence the NRF2-ARE pathway through the recruitment of the histone deacetylase3 (HDAC3), MAFK hypoacetylation [146,147,148], and a direct interaction between HDAC3 and MAFK, preventing any interaction with NRF2 and thus any NRF2 transcriptional facilitation [149]. Based on NRF2-deficient mouse experiments, a more pronounced NF-κB activity is observed, leading to a low inducible HO-1 expression, ROS accumulation, and increased inflammatory response [150,151,152].

## 4. NRF2 and Brain (Dys)Functions

### 4.1. NRF2 in Brain Control of Energy Metabolism

NRF2 has been described to play a role in obesity and metabolism [153] (Table 2). Following cellular stresses, the NRF2 pathway is activated to stimulate defence mechanisms, reduce body weight, and increase energy expenditure. Jardim and colleagues recently described that young mice, exposed to a high-caloric diet during their early-life period, had learning and memory impairments [154]. The authors revealed that this specific diet induced an oxidative stress in the hippocampus, a brain region known to have critical functions in cognition and memory but also to play certain roles in regulating food intake [155]. A high-caloric diet reduces NRF2 recruitment, whereas a dietary energy restriction triggers its activation and thereby induces many beneficial effects on health [156,157].

Other recent studies show that NRF2 is involved in brain control of food intake [158]. The hepatokine FGF21 is an endocrine signal, whose blood level increases during fasting, protein restriction, or high carbohydrate intake [159,160,161,162]. At the molecular level, FGF21 can stimulate the nuclear translocation of NRF2 and its recruitment to specific DNA regions that contains ARE sequences, triggering the transcription of the Oxt gene in oxytocin (OXT) neurons. These OXT neurons promote fat intake and limit sugar intake [158]. Thus, the FGF21-NRF2-oxytocin axis is viewed as an integrative system that contributes to the macronutrient-based diet selection, shifting from sugar to fat preference as an adaptive response to changes in the metabolic state. Parallel FGF21-sensitive glutamatergic neuronal pathways coexist to suppress carbohydrate intake independently of OXT neurons [163]. Through upregulation of NRF2 levels, an FGF21 administration may also show positive effects by protecting against diabetes-induced blood-brain-barrier disruption [164,165]. Inversely, in periphery, NRF2 regulates hepatocyte FGF21 secretion [166,167]. Interestingly, activation of NRF2 in hepatocytes in obese mice represents a strategy to stimulate FGF21 release in the blood, which decreases lipogenesis in the liver, promotes lipolysis in the white adipose tissue, and stimulates thermogenesis in the brown adipose tissue, thus preventing obesity and associated hepatosteatosis [167]. 

**Table 2:** NRF2 and brain control of energy metabolism.

Characteristics	NRF2 Pathway Status	Key Points	References
Fasting (in mice)	downregulation in the brain	reduced FGF21 sensitivity, reduced oxytocin signaling, increased sugar preference	[158]
Sugar intake (in mice)	activation in the brain	increased FGF21 sensitivity, activated oxytocin signaling, reduced sugar preference	[158]
Diabetes (in db/db diabetic mice)	downregulation in the brain	correlation with blood-brain-barrier permeability	[164,165]
	downregulation in the brain reversed by FGF21 treament	blood-brain-barrier permeability reversed by FGF21 treament	[165]
Diabetes, obesity (*Trsp*^RipKO^ mice)	downregulation in the brain	reduction in anorectic POMC neurons, loss of leptin sensitivity	[168]
	downregulation in the brain reversed by Keap1 invalidation	reduction in anorectic POMC neurons, loss of leptin sensitivity reversed by Keap1 invalidation	[168]

The role of NRF2 in control of brain energy homeostasis has been further demonstrated in diabetic animals, in which diabetes has been induced by genetic Trsp deficiency [168]. The Trsp gene encodes the tRNA for selenocysteine (tRNA^sec^), which is essential for the biosynthesis of selenoproteins, a group of proteins that contain selenocysteine residues [169], such as GPX and thioredoxin reductase. Trsp deletion in hypothalamic cells depletes selenoproteins in the hypothalamus, which provokes a local oxidative stress. This cellular stress alters and probably kills anorectic pro-opiomelanocortin (POMC) neurons, reducing leptin sensitivity in the hypothalamus, a crucial step in the pathogenesis of the metabolic disorder [168]. Induction of NRF2 in this model prevents oxidative damage in POMC neurons and reduces metabolic impairments.

These studies suggest that stimulation of NRF2 activity in the brain is a way to ameliorate whole-body energy homeostasis. Interestingly, NRF2 activity can be modified by daily consumption of specific food and phytochemicals. Most of these micronutrients/compounds are contained in plant-based food such as kaempferol (a polyphenol found in some fruits and vegetables), niga-ichigoside (a triterpenoid saponin from rosacea), salacia chinensis extracts (a climbing plant that contains triterpenes, phenolic compounds, and glycosides), hydroxytyrosol (the main phenolic compound in olives), curcumin, rebaudioside (a sweetener from Stevia rebaudiana), chicoric acid, and omega 3 and polyunsaturated fatty acids [170,171,172,173,174,175,176,177,178]. In addition, *NFE2L2* gene expression can be upregulated by fibre-enriched diets [179]. Effects of these nutritional interventions on NRF2 were found in peripheral organs, and action on brain remains to be determined. It also remains to be established whether effects of these food-derived compounds on NRF2 are direct and/or associated with a better overall health.

### 4.2. NRF2 in Brain Ageing

Humans are among the longest-living mammals, with a maximum lifespan potential (MLSP) of 100 years. In animal models, the naked mole rat represents a unique model of healthy ageing, with a MLSP of 31 years [180]. NRF2 activity correlates with the MLSP of these naked mole rats. Indeed, it has been reported that long-living animal species have higher NRF2-signalling levels, highlighting the importance of NRF2 protection against ageing and ageing-related diseases [180].

In normal ageing, NRF2 activity, as well as expression of NRF2 target genes, decrease with age in many organs, including the brain [181,182] (Table 3). Consequently, the antioxidant/oxidant equilibrium is disrupted during brain ageing, thus favouring oxidative stress and associated damage. From a general point of view, the age-related progressive decline in NRF2 activity has been attributed to decreased expression of its positive regulators (PI3K, p62, CBP), increased expression of its negative regulators (KEAP1, BACH1), and also a general decrease in NRF2 protein expression itself [183,184,185]. As a result of NRF2 activity decline during ageing, GSH content as well as GSH- and antioxidant-related enzyme activity decrease in rat brain, while mitochondrial production of superoxide anions gradually increases over time [186,187]. These data obtained from animal experiments have been confirmed in a few human cohort studies, in which oxidative stress appears to be strongly linked to cognitive decline during ageing [188,189,190,191].

Multiple studies show that NRF2, whose activity is reduced during ageing, can be considered as a targetable pathway [181,182,192,193]. Thus, the activation of NRF2 in ageing could theoretically be beneficial. However, one recent study reports that the effect of knocking out NRF2 in old mice is rather contradictory to this conclusion [194]. Han and colleagues described that 18-month-old NRF2-KO mice had significantly better motor skills, less apoptosis in substantia nigra (SN), more tyrosine hydroxylase (TH)-positive signals in the striatum, a reduction in the 4-HNE staining in dopaminergic neurons of SN, and decreased iron accumulation in various brain regions compared to WT mice. Han and colleagues claimed that the regulation of brain iron metabolism in these old NRF2-KO mice was mediated by a decrease in FPN1 level, the only known exporter of non-heme iron in mammals, in brain endothelial cells, thus limiting the entry of iron into the brain and the subsequent formation of harmful reactive species, such as HO^●^. This NRF2-KO mouse model, however, presents collateral damages with an exacerbation of the dysregulation of iron metabolism, an increase in ROS, and higher MDA levels in liver and spleen [195]. The results obtained by Han and colleagues contradict other studies. Indeed, the downregulation of ferroportin 1 in hippocampus of AD mouse models and also AD patients is associated with brain atrophy and cognitive impairment [196]. More recently, ferroportin loss in ageing brains has been described to be a key reason for iron mismanagement, and ferritin accumulation induced protein aggregation in Down syndrome, dementia, Alzheimer’s disease, and Parkinson’s disease (PD) [197]. The above studies claim that restoring Fpn expression, an NRF2 target gene coding for FPN1, in ageing and neurodegenerative models will ultimately result in a decrease in ferroptosis, protein aggregation, and memory loss.

**Table 3:** NRF2 and ageing.

Characteristics	NRF2 Pathway Status	Key Points	References
Normal ageing	decrease with age in many organs, including the brain	disruption of the antioxidant/oxidant equilibrium	[181,182]
Normal ageing	increased activity in liver of long-lived rodents	increased NRF2:ARE binding and antioxidant enzymatic activities	[183]
Normal ageing	decrease with age in the brain	decline in antioxidant enzyme activity after 12 months and incraeased superoxide anion in mitochondria	[186,187]
Normal ageing	possibly decreased based on blood levels of lipoperoxidation	indication of higher oxidative stress associated with cognitive decline	[188]
Normal ageing	possibly decreased based on blood levels of antioxidant enzyme	indication of lower antioxidative response associated with cognitive decline	[189]
Normal ageing	not directly implicated	increased oxidative stress associated with increased morbidity	[190]
Normal ageing	possibly decreased based on blood levels of antioxidant enzyme	indication of lower antioxidative response associated with cognitive decline	[191]
Normal ageing upon NRF2 deletion	inactive due to NRF2 knockout in mouse	reduced midbrain oxidative stress and reduced motor dysfunction in aged mice	[194]
Normal ageing upon NRF2 deletion	inactive due to NRF2 knockout in mouse	increased oxidative stress in liver and spleen	[195]
Cell culture model of HGPS	decreased due to sequestering of NRF2 by progerin	oxidative stress and aging defects in HGPS depend on NRF2 activity	[198]

Premature ageing is a hallmark of Hutchinson–Gilford progeria syndrome (HGPS), a rare genetic disorder. De novo mutation in the LMNA gene induces the expression of progerin, a truncated lamin A protein that triggers various cellular defects. NRF2 has been recently identified to be one of the progerin’s targets [198]. Using human skin fibroblasts containing GFP-progerin, Kubben and colleagues revealed that progerin sequesters NRF2 and causes its mislocalisation to the nuclear periphery, altering its transcriptional activity and promoting oxidative stress. Interestingly, introducing constitutively active NRF2 to the HGPS fibroblast model restored most of the ageing-associated defects and showed the potential of the NRF2 pathway as an ageing modifier.

A deterioration of NRF2 activity is a critical point in the ageing process as well as age-related diseases. In order to restore the oxidative balance, many antioxidant supplementation therapies (e.g., antioxidant vitamins, coenzyme Q, resveratrol, curcumin) have been implemented. However, tests of the effectiveness of such treatments in humans have raised contrasting results [199,200,201]. Some NRF2-activating compounds have been described as potential senotherapeutic drugs. There are two kinds of senotherapeutics: senolytics, which induce cell death (also called senolysis) of senescent cells (e.g., curcumin, quercetin, berberine), and senomorphics, which suppress pro-inflammatory senescence-associated secretory phenotypes produced by senescent cells (e.g., rapamycin, metformin, epigallocatechin gallate) [202,203,204,205,206]. A promising avenue, increasingly raised in the literature, would then be to target the senescence process. Cellular senescence, one of the hallmarks of ageing, is characterized by the attainment of a quiescent state where cells lose their ability to divide and resist cell death. In the brain, the proportion of senescent glial and neuronal cells increases with age [207] and could be implicated in the onset or aggravation of cognitive impairment [208]. Many preclinical studies show positive effects of senolytics, which notably improve cognitive abilities in rodents and thus reduce the symptoms of age-related diseases [209,210]. Therefore, NRF2 can play a key role by regulating the multiple senescence-associated pathways [211]. The re-activation of NRF2 might thus be able to control and delay the ageing process. It remains to be evaluated how and when targeting NRF2 in ageing could be beneficial or detrimental. Key points regarding the role of NRF2 in ageing have been detailed below in Table 3.

### 4.3. NRF2 in Age-Related Neurodegenerative Disorders

In recent years, many findings describing the central role of the NRF2 transcription factor in redox homeostasis and anti-inflammatory functions in neurodegenerative disorders (NDDs) have emerged [212] (Table 4). As mentioned above (Section 3.2), brain NRF2 expression is found to be higher in glial cells (astrocytes and microglia) than in neurons, whereas the major NRF2 repressor, KEAP1, appears to be equally expressed in all brain cells. We can then postulate that glial cells are able to protect neurons against oxidative damage. As life expectancy is increasing, the prevalence of neurological diseases is likely to rise accordingly. Age-dependent cognitive loss as well as NRF2 activity decline are observed in NDDs such as AD and PD [213,214,215].

NRF2 appears to be an ideal target to modulate inflammation, autophagy, proteostasis, and oxidative stress in NDDs. Animal models helped to partially dissect the pathological mechanism in neurodegenerative diseases. As proof, the ablation of NRF2 in APP/PS1 mouse model leads to an increase in the beta-amyloid (Aβ) level (without increasing the number of Aβ plaques), pro-inflammatory molecules (production of IFNγ, IL-1β, IL-6, and TNF-α), oxidative DNA damage (increase in 8-hydroxydeoxyguanosine, 8-OHdG), glial cells reactivity (increase in Iba1 and GFAP staining), defective neuronal autophagy, and exacerbation of memory and cognitive defects in 11-to-12-month-old transgenic mice [120,216,217]. Knocking out NRF2 in a bigenic AD mouse model (AT-NRF2-KO) with combined amyloidopathy (expression of human mutant hAPPV717I) and tauopathy (expression of human mutant hTAUP301L protein) [119] also promoted inflammatory and oxidative stress. These AT-NRF2-KO mice have higher cognitive impairments and lower hippocampal long-term potentiation (lower electrophysical capacity of hippocampal neurons) already at six months of age before the appearance of Aβ and Tau pathologies.

The lentivirus-mediated expression of NRF2 in hippocampi of 9-month-old APP/PS1 mice led to an increase in insoluble Aβ, but it also led to attenuation of astrocyte reactivity, higher induction of HO-1 (neuroprotection), and reduction in spatial learning impairments [218]. The analysis of the astrocyte translatome, obtained by translating ribosome affinity purification sequencing (TRAP-seq), of the two AD mouse models, APP/PS1 and MAPTP301S mice, showed partial overlap of gene expression levels with age-altered pathways [219]. Jiwaji and colleagues crossed a GFAP-NRF2 mouse strain, mice overexpressing NRF2 in astrocytes, with APP/PS1 or MAPTP301S mice. NRF2 overexpression in APP/PS1 astrocytes reduced amyloid pathology as well as glial cells reactivity (decrease in Iba1 and GFAP expression) and reversed cognitive deficits observed in APP/PS1 mice. Cortical neurodegeneration and Tau pathology were decreased in MAPTP301S mice crossed with GFAP-NRF2 mice compared to the MAPTP301S mice. Some other studies report that NRF2 nuclear expression levels, in neurons and astrocytes, were decreased in AD and AD variant with Lewy bodies in CA1 of the hippocampus or cortical regions compared with age-matched control cases [120,220]. Thus, manipulation of astrocyte response through NRF2-associated pathways represents a promising therapeutic opportunity in AD.

An unbalance of NRF2 expression was demonstrated in autopsy samples of AD and PD patients. However, nuclear NRF2 staining was preserved, even amplified, in PD substantia nigra neurons [220,221] but insufficient to protect these neurons from degeneration. The expression of a PD-linked gene encoding α-synuclein (α-syn) in dopaminergic neurons of Drosophila [222], overexpression of human wild-type α-syn within murine SNpc [223], and toxin-based models by injection of 6-hydroxydopamine [224] and 1-methyl-4-phenyl-1,2,3,6-tetrahydropyridine [212,223,225,226] were used to mimic in vivo PD. In such models, the authors described a decreased locomotor activity, a progressive neuronal loss (TH-positive neurons), an intense astrogliosis and microgliosis, and a decrease in NRF2 activity.

Anandhan and collaborators described that NRF2 knockout in a PD mouse model (overexpression of human α-synuclein) resulted in an increase in the PD pathology associated with a behavioural dysfunction [126]. Indeed, at 3 months of age, hα-Syn+/NRF2-/- mice showed increased cognitive defects. At molecular and cellular levels, authors showed higher levels of phospho-α-synuclein, oxidation of lipids (increase in 4-HNE), increase in pro-inflammatory gene expressions (*Nos2*, *Ptgs2*), and increases in microglial activation and autophagy, especially in the midbrain and striatum. They also found a higher loss of tyrosine hydroxylase-positive neurons in nigrostriatal regions. An overexpression of human alpha-synuclein in the ventral midbrain of NRF2 knockout mice via the stereotaxic delivery of a viral vector leads to a more severe loss of dopaminergic neurons, inflammation, and microglia activation [227]. In this study, microglia were particularly altered toward pro-inflammatory/low phagocytotic profiles, showing again the important role of the induction of the NRF2 pathway in glial cell responses in NDDs.

All these results support new approaches in which targeting the NRF2 pathway could prevent and/or delay tissue injury. This has been notably confirmed by the use of celastrol. This natural bioactive ingredient derived from the Tripterygium wilfordii Hook F plant, described to activate the NRF2 pathway, decreases neuronal death, attenuates neuroinflammation, and relieves motor deficits [223] in PD mouse models. It was also described by Zhang and colleagues that celastrol could inhibit the NLRP3 inflammasome pathway, a critical component of the innate immune system, leading to caspase-1 activation and pro-inflammatory cytokines (IL-1β and IL-18) secretion in these PD mouse models. Many other studies have clearly shown that NRF2 overexpression led to beneficial effects in brain pathologies. Thus, astrocyte-specific overexpression of NRF2 delays motor deficits and synuclein aggregation in an A53T α-syn mutant mouse model [228], delays neurodegeneration in amyotrophic lateral sclerosis mouse models [229], and protects against optic tract damage and behavioural alteration in a mouse model of cerebral hypoperfusion [230]. Beneficial effects are also described in a mouse model with a fatal neurodegenerative disorder, called Alexander disease, caused by dominant mutations in the astrocyte intermediate filament glial fibrillary acidic protein leading to astrocyte overactivation [231]. All these preclinical data validated that NRF2 upregulation has neuroprotective effects.

**Table 4:** NRF2 and NDDs.

Characteristics	NRF2 Pathway Status	Key Points	References
AD mouse model APP/PS1	genetic ablation of *Nfe2l2*	increase in the amyloid level, oxidative and inflammatory markers, autopahgy, gliosis, and cognitive impairments	[120,216,217]
AD mouse model APP/PS1	lentivirus-mediated expression of NRF2 in hippocampi	increase in insoluble Abeta, decrease in astrogliosis, higher HO-1 level, reduction in spatial learning impairments	[218]
AD mouse model bigenic APP/TAU	genetic ablation of *Nfe2l2*	increase in oxidative and inflammatory markers, cognitive impairments, and lower LTP	[119]
AD mouse model APP/PS1	crossed with GFAP-NRF2, overexpression in astrocytes	reduction in amyloid pathology, gliosis, and cognitive deficits	[219]
AD mouse model MAPTP301S	crossed with GFAP-NRF2, overexpression in astrocytes	reduction in Tau pathology, cortical neurodegeneration	[219]
AD and AD variant with Lewy bodies	decreased NRF2 levels	hippocampus, cortex	[120,220]
PD	preserved or amplified NRF2 levels	substantia nigra neurons	[220,221]
α-syn and toxins PD models	decreased NRF2 levels	gliosis, neuronal death, locomotor impairments	[212,223,225,226]
α-syn PD mouse model	genetic ablation of *Nfe2l2*	higher levels of P-syn, inflammation, microgliosis, and autophagy, loss of TH neurons, increase in cognitive impairments	[126]
A53T α-syn PD mouse model	crossed with GFAP-NRF2, overexpression in astrocytes	motor deficits delayed	[227]
Amyotrophic Lateral Sclerosis (ALS)	crossed with GFAP-NRF2, overexpression in astrocytes	neurodegeneration delayed	[228]
Alexander’s disease	NRF2 overexpression in astrocytes	decrease in GFAP expression and Rosenthal fibres, restoration of body weight	[231]

Observations from post-mortem brains of PD patients reveal that oxidative stress may be implicated in the pathogenesis of PD [232,233]. This has been confirmed by an increase in oxidative damage markers (8-OHdG, MDA, 4-HNE) with decreased antioxidant defences in the brain and the blood of PD patients [234,235,236]. As a regulator of cellular antioxidant response, the NRF2 pathway appears as a potential target to prevent and/or delay tissue damage in PD. Indeed, results obtained from manipulation of the NRF2 pathway in animal models highlight the relevance of considering NRF2 as a potent modulator of neurodegeneration. Key points on the role of NRF2 in NDDs are resumed in Table 4.

### 4.4. NRF2 in Brain Cancer

Primary brain tumours, especially the heterogeneous group of diffuse gliomas, are still among the most devastating clinical conditions for which no cure exists in most cases. Understanding of how exactly those tumours either arise or infiltrate the brain is still incomplete to date. As the NRF2 pathway is involved in multiple key pathways in humans, it is worth having a closer look at its potential implication in brain tumorigenesis as well as its suitability as a treatment target. As oxidative stress is supposed to play a role in the development of high-grade gliomas, it was obvious that NRF2, as a central regulator of cell stress signalling, could be implicated (Table 5).

In the early days of brain tumour research related to the role of NRF2, it could be demonstrated in certainly oversimplified in vitro cell culture models that the downregulation of NRF2 led to increased levels of both apoptosis and autophagy [237,238]. The results obtained from more complex in vivo murine mouse models met those expectations by showing that reduced NRF2 levels, due to either direct downregulation of NRF2 or blocking its upstream activators ERK and PI3K, were associated with reduced glioma cell proliferation, enhanced apoptosis and ferroptosis, and impaired angiogenesis [239,240,241]. The anti-angiogenic effect of NRF2 could be linked to a decrease in hypoxia-inducible factor 1α (HIF-1α) stabilization, with a subsequent decrease in vascular endothelial growth factor (VEGF) expression [242]. A strong emphasis was also put on the positive effect that NRF2 exerted on the self-renewal capacity of glioma cells, as at that time, the maintenance of glioma stem cell properties was considered as a key factor in treatment resistance [121]. In the clinical context, NRF2 gained further attention, as it could be demonstrated that NRF2 expression was a negative prognostic factor for patient survival in glioma cohorts [243,244]. The negative association of NRF2 expression and glioblastoma patient survival could be later corroborated in a large cohort of The Cancer Genome Atlas (TCGA) and be linked mechanistically not only to its already described proliferation-enhancing properties but also to an invasion-stimulatory effect in the context of positive feedback between the autophagy-associated proteins SQSTM1/p62 and NRF2 [245]. Of note, gliomas with isocitrate dehydrogenase 1/2 (IDH1/2) mutations display significantly lower NRF2 levels as compared to their IDH1/2 wildtype diffuse glioma counterparts; however, in the subclass of IDH-mutant gliomas, high NRF2 levels were associated with a worse clinical prognosis [246,247]. IDH wildtype glioblastoma often display increased human telomerase reverse transcriptase (hTERT) activity in both hTERT wildtype and mutation contexts that can be stimulated by NRF2, thereby preventing ROS-induced glioma cell death [248].

A similarly detrimental clinical situation to the one of diffuse high-grade glioma is often encountered in patients with brain metastases. Most recently, it could be shown that even in early stages of non-small-cell lung cancer (NSCLC), high NRF2 expression levels in cancer cells were associated with an increased risk of developing brain metastases [249]. This NRF2 overexpression in more malignant clinical situations seems to be strongly related to different mutations in the NRF2-KEAP1-ARE cell survival pathway [250]. In most patients with NSCLC brain metastasis, those mutations were either specific or increased in brain metastases as compared to the primary tumours. The mutations either affect the capacity of KEAP1 to induce the degradation of NRF2 or alter the Neh2 domain of NRF2 that is responsible for binding KEAP1 constituting a prerequisite for subsequent degradation. Several mutations specifically led to NRF2 stabilization and nuclear translocation, with subsequent increased activation of its target genes. In the same study, the authors even went beyond studying NSCLC and detected different NRF2 mutations also in circulating tumour cells from patients with melanoma, breast, and colon carcinoma metastases [250]. In addition to the negative primary effects that could be attributed to NRF2 signalling, similarly detrimental functions were reported in the treatment context of brain cancer [251,252].

**Table 5:** NRF2 and cancer.

Characteristics	NRF2 Pathway Status	Key Points	References
Glioma cell line	downregulation	increased apoptosis	[237]
	downregulation	increased autophagy	[238]
	overexpression	reversed ERK and PI3K-inhibition-induced inhibition of cell viability	[239]
	overexpression	increased proliferation, resistance to ferroptosis, increased oncogenic potential	[240]
	downregulation	reduced proliferation	[241]
	downregulation	reduced proliferation, reduced mitochondrial oxygen consumption	[242]
	downregulation	reduced proliferation	[121]
	upregulation	increased proliferation, tumour cell infiltration, and mesenchymal transition	[245]
	downregulation	decreased resistance towards chemotherapy	[247]
	overexpression	prevention of ROS-induced cell death	[248]
	downregulation	enhanced sensitivity towards chemotherapy and irradiation	[251]
	overexpression	increased cell survival after chemotherapy	[252]
	overexpression	reduced ROS levels, increased increased cell survival	[253]
Human glioma tissue	upregulation (as compared to normal tissue)	potentially implicated in glioma progression	[239]
	upregulation	worse patient survival	[240]
	upregulation (as compared to normal tissue)	potentially implicated in glioma progression	[241]
	upregulation	worse patient survival	[241]
	upregulation	increased angiogenesis	[242]
	upregulation with grade of malignancy	potentially implicated in glioma progression	[243]
	cytoplasmic expression	association with worse prognosis	[243]
	nuclear expression	association with better prognosis	[243]
	overexpression	association with worse prognosis, more tissue necrosis, correlation with high HIF1alpha levels	[244]
	overexpression	decreased progression-free survival	[245]
	overexpression	worse patient prognosis in anaplastic glioma	[246]
	overexpression	upon irradiation and chemotherapy	[251]
	overexpression	association with worse prognosis	[252]
Xenograft glioblastomas mouse model	downregulation	reduced tumour growth, reduced proliferation, increased apoptosis, reduced angiogenesis	[241]
	downregulation	reduced VEGF expression, reduced angiogenesis	[242]
Human lung carcinoma tissue	overexpression	higher risk of brain metastasis	[249]
	increased mutational rate of NRF2 pathway	higher risk of brain metastasis	[250]

Both chemotherapy and irradiation treatment enhanced NRF2 levels in human glioma cell lines and patient samples, and the degree of NRF2 expression was associated with earlier recurrences. A more recent study was able to show that NRF2-related protumorigenic properties in gliomas are not exclusively linked to ROS regulation or metabolic adaptation but are also linked to an enhanced expression of the transcriptional coactivator TAZ belonging to the HIPPO pathway, most probably by activating functional enhancers in the regulatory regions of TAZ [253]. As an upstream regulator of NRF2-mediated ROS tolerance in malignant glioma cells, the Wiskott–Aldrich syndrome protein (WASP)-interacting protein (WIP) inhibits KEAP1, which acts as an NRF2 repressor, finally leading to reduced ROS levels and unwanted glioma cell survival [253]. In summary, there is a large body of evidence that increased NRF2 levels are associated with (a) various tumour-promoting functions in malignant gliomas, (b) negative patient survival in diffuse gliomas and glioblastomas, (c) tumour cell senescence [254], and (d) resistance towards radio-chemotherapy. 

Targeting NRF2 has mainly been restricted to preclinical cell culture and animal studies, while promising early clinical concepts are lacking. This might be related to the fact that, over the last several decades, pharmacological research regarding NRF2 modulating drugs has focused on NRF2-activating rather than -inhibiting drugs, with the primary goal to enhance cellular tolerance towards ROS, which might be beneficial in a plethora of degenerative disorders but is probably less promising as a novel concept in tumour treatment [255]. The major challenge in potential novel treatment approaches blocking NRF2 in cancer and especially in malignant gliomas will be to overcome expected severe negative side effects in residual cells, most probably neurons and muscle cells, which both may rapidly suffer from a decreased capacity to deal with high ROS levels, thereby being at risk to develop secondary neurodegenerative or myopathic symptoms.

## 5. Epigenetic Regulation of NRF2 Signalling

### 5.1. NRF2-Mediated Transactivation and Chromatin Level Regulation

The basic unit of chromatin is the nucleosome, which consists of eight histone proteins that are surrounded by ~147 bp fragments of DNA. These nucleosomes progressively condense to form chromatin [256]. Chromatin can be found in two main configurations: heterochromatin, which is highly compacted and has low accessibility for transcription factor binding; and euchromatin, which is more open and therefore where most gene activation and transcription occurs [257]. Promoters and enhancers are the regions of DNA that control gene expression. To initiate the transcription of a specific gene, it is necessary for transcription factors (TFs) to bind to the promoter of that gene, recruiting the rest of the machinery required for transcriptional activation (other TFs, cofactors, and RNA polymerase II holoenzyme (RNA Pol II)). Furthermore, enhancer elements are regulatory regions that interact with promoters and transcriptional machinery to stabilize the RNA Pol II complex and increase gene expression [258]. While the function of promoters depends on their position and orientation relative to the target gene, enhancers can interact with promoters located far away in chromatin due to three-dimensional loop formation [259,260].

Histone acetylation increases the accessibility of chromatin for transcription factor binding, which often links to gene transcription activation. On the other hand, deacetylation usually leads to gene repression. Histone acetyltransferases (HATs) and HDACs are the proteins that acetylate or deacetylate histones, respectively. Reduced NRF2 expression is associated with decreased histone acetylation at *NFE2L2* promoter, and HDAC inhibition can increase NRF2 expression with beneficial effect in glial cells [261,262]. In contrast, histone methylation is associated with both gene repression and activation, depending on the residue that is methylated and the degree of methylation. Histone methyltransferases and histone demethylases are the proteins that methylate or demethylate the histones, respectively. Histone H3 lysine 27 trimethylation (H3K27me3) is a histone modification associated with Polycomb-mediated gene repression and found to be deposited at the *NFE2L2* promoter by EZH2 enzyme of the PRC2 complex in lung cancer cells [263]. Thus, Polycomb-mediated repression of *NFE2L2* might also occur in the different cell types of the brain.

Once NRF2 is released from KEAP1-mediated retention in the cytoplasm, it translocates to the nucleus and, after binding to ARE sequences on DNA, regulates the transcription of its target genes in collaboration with sMAF proteins (Figure 3). This process is called NRF2-mediated gene transactivation. ARE sequences are found in the promoters of NRF2 target genes, but there is also evidence of ARE sequences located at enhancer elements [264,265]. Typically, after NRF2 binds to the promoters of its target genes, recruitment of other TFs or cofactors is required. Some examples are FOS, MAFB, LHX3, MEF2A [265], ATF4 [266], or BRG1 [267]. It is also known that ARE-like sequences can be recognized by other Cap’n’Collar TFs, including BACH1 and BACH2 [56] and members of the AP1 complex [268]. A defining feature of TF-mediated transactivation is the recruitment of the Mediator complex that creates the physical interaction between the TF and RNA Pol II. In case of NRF2, the interaction takes place with a Mediator subunit called MED16 [269]. Moreover, there are studies showing that NRF2 can also associate with other transcriptional regulators such as chromodomain helicase DNA-binding protein 6 [270], receptor-associated co-activator 3 [271], and NAD^+^-dependent histone deacetylase sirtuin 6 [272]. In addition to protein-coding genes, NRF2 can also activate the expression of long non-coding RNAs after binding to their promoters [273]. 

The gene activation by NRF2 is accompanied by the recruitment of histone acetylases that increase local histone acetylation. This can be counteracted by activity of other factors such as NF-κB by depriving NRF2 of its co-activator and facilitating histone deacetylation via recruitment of histone deacetylases such as HDAC3 [149]. There is some evidence that NRF2 can repress the expression of a variety of target genes. This repression by NRF2 can be direct, through the formation of inhibitory complexes with other transcription factors [274], or indirect, for example, through the activation of the expression of microRNAs (miRNAs) [275]. Some examples of miRNAs that can be activated by NRF2 are miR-365-1/miR-193b cluster, miR-29-b1, and miR-125-b1 [264,276]. In line with this, studies such as the one by Kwak and collaborators indicate that NRF2 is primarily an activator and blocks gene expression indirectly [277].

The overall transcriptomic response to NRF2 activation depends on the presence of accessible ARE-containing enhancers available for NRF2 binding. The epigenetic mechanisms controlling enhancer accessibility at the chromatin level include DNA methylation, histone modifications, non-coding RNAs, and chromatin remodelling [278]. A large part of the accessible chromatin is determined by the cell type [279,280], but it is also determined by environmental exposures and genotype of the cell in question [281,282]. Through genome-wide chromatin immunoprecipitation analysis, thousands of NRF2 binding sites have been identified in vivo [283], with a large proportion of AREs showing cell-type-selective binding. As an interesting example, NRF2 binding sites at target genes associated with poor survival across multiple cancers have a particularly permissive chromatin structure across cell types, allowing broad activation of these genes [284]. Meanhile, other NRF2 targets, not associated with cancer, show more cell-type-selective chromatin accessibility at their ARE sites. Despite being a biomedically relevant target tissue, the occupied NRF2 binding sites in the different cell types of the central nervous system have not yet been extensively studied. However, as described below, this could yield important insights into the cell-type-specific activity of NRF2.

As mentioned above, the availability of AREs for NRF2-mediated activation is also controlled by competitive binding by BACH1 and BACH2 to the same sequence motif [53,54,55,56] (Figure 3). BACH1 and BACH2 are predominantly transcriptional repressors, thereby counteracting the NRF2-mediated activation [285,286] and possibly also inducing chromatin compaction at the AREs. Thus, the final transcriptomic response to NRF2 activation is determined by not only the array of available ARE-containing enhancers but also the balance between NRF2 and BACH1/2 expression levels in the target cell.

### 5.2. Different NRF2 Activity in Brain Cell Types and Approaches for Cell-Type-Specific Targeting

As for any gene, there are multiple determinants for *NFE2L2* expression levels. Firstly, its promoter contains ARE sequences, allowing for auto-regulation through NRF2 binding [287] (Figure 3). In addition, a xenobiotic response element for aryl hydrocarbon receptor, NF-κB, and AP2 binding sites can be found at the promoter [287,288,289], among others. Moreover, KRAS signalling is known to induce NRF2 expression in the context of cancer [290].

Across brain cell types, *NFE2L2* is the least expressed in different neurons, while high expression can be detected in non-neuronal cell types [111,291]. More specifically, the highest expression levels were detected in microglia and astrocytes, consistently with the above-discussed reliance of neurons on glial cells for their antioxidant response and neuroprotection [292,293,294]. The neuronal repression of NRF2 was found to be accompanied by histone H3 hypoacetylation at the *NFE2L2* promoter and could be reversed by HDAC inhibitors in neurons [295]. The repression was induced during neuronal differentiation and maturation, leading to impaired antioxidant response, but could not be reversed by HDAC inhibition in very young neurons, suggesting an important developmental role. Indeed, the NRF2 repression was proposed to be necessary for normal neurodevelopment due to its interference with WNT signalling activation. Interestingly, in parallel with low expression of NRF2, midbrain dopaminergic neurons are enriched for high BACH2 levels [111], allowing further repression of NRF2 target sites in neurons and ensuring correct neuronal commitment. Given that *NFE2L2* promoter hosts an ARE, it would be interesting to investigate whether deacetylation of the promoter is induced by increasing BACH2 binding during neurogenesis. Finally, in addition to transcriptional control, *NFE2L2* mRNA was suggested to be targeted by multiple miRNAs in neuronal SH-SY5Y cell line, possibly further contributing to the low NRF2 expression in neurons [296].

While neurons are lacking in their own NRF2 signalling, glial cells and brain tumours derived from them, such as glioblastomas, show high NRF2 activity that is associated with a poor prognosis (Section 4.4). Therefore, depending on the pathology and the cell type affected, either further activation of NRF2 signalling, as in neurodegenerative diseases and ageing, or repression of NRF2 signalling, as in cancer, would be needed. For example, activation of NRF2 using compounds such as dimethyl fumarate can have beneficial effects in human and mouse models on both AD and PD [119,297,298,299,300,301,302]. Similarly, in some cancer models, an inhibition of NRF2 by small compounds has shown promise in in vitro assays [303]. However, the existing direct activators or inhibitors of NRF2 signalling often have high turnover and are difficult to deliver to the target tissue, creating further challenges for systematic treatments. Furthermore, as discussed above, systematic ablation of NRF2 activity can lead to side effects in other tissues such as spleen and liver [195]. 

The cancer-related hyperactivity of NRF2 signalling drives an overexpression of target genes located in permissive chromatin [284] but, in some cancers, such as non-small-cell lung cancer, also enhances reprogramming revealing novel cancer-specific ARE targets for NRF2 binding occurs [304]. Similarly, cell-type-specific profiles of ARE-containing enhancers are likely to exist in the different cell types of the brain and brain tumours [305,306,307]. Genome-wide mapping of ARE-carrying enhancers targeted by NRF2 and/or BACH1/2 in the different cell types of the brain and various brain tumours and understanding the contribution of those target genes to different pathologies might allow for more precise approaches for targeting NRF2 signalling. Indeed, analysis of BACH1 regulation in ventral midbrain found BACH1 target genes to be involved in several neuroprotective pathways but repressed during neurodegeneration [308]. Interestingly, inhibition of BACH1 enabled an NRF2-mediated increase in the target genes’ expression in neuronal cells and protected against loss of dopaminergic neurons in a mouse model of PD. Whether a similar approach could be taken with BACH2, which shows a more neuron-specific expression profile [111], would be interesting to test. In addition, other signalling pathways such as retinoic acid and oestrogen signalling are known to interfere with NRF2 signalling [309] and provide an alternative avenue for controlling the pathway. However, these approaches also require a more detailed understanding of the cell-type-specific target genes. With the arrival of low-input and single-cell technologies, rare cell types of the brain can now also be studied [310].

Finally, more detailed knowledge of the genome-wide action of TFs such as NRF2 and BACH1/2 is beneficial not only for better targeting of NRF2 signalling but also for diagnostics and better understanding of disease risk. Indeed, disease-associated regulatory genetic variants are known to be enriched in cell-type-selective enhancer regions, where they can disrupt or create TF bindings sites [311,312,313]. Through cell-type-specific alterations in gene expression, such variants can then influence disease risk and, when well understood, be used for diagnostic purposes. This is also true for enhancers bound by NRF2: Wang and collaborators identified several disease-associated single nucleotide polymorphisms capable of disrupting AREs and leading to allele-specific NRF2 binding [283]. Interestingly, a variant with strong association with parkinsonian disorders was found to decrease NRF2 binding at an ARE in the intron of the Microtubule Associated Protein Tau (MAPT) gene and to drive lower MAPT transcription in neuronal progenitors. This provides the first insight into the mechanistic explanation for this variant’s association with PD and related disorders.

## 6. Conclusions

Oxidative stress and inflammation are the two most common features of brain diseases. Here, we provided evidence that NRF2, by its anti-oxidant and anti-inflammatory properties, could be a promising therapeutic target for the maintenance of brain functions as well as for brain dysfunctions improvement.

**Figure 4:**
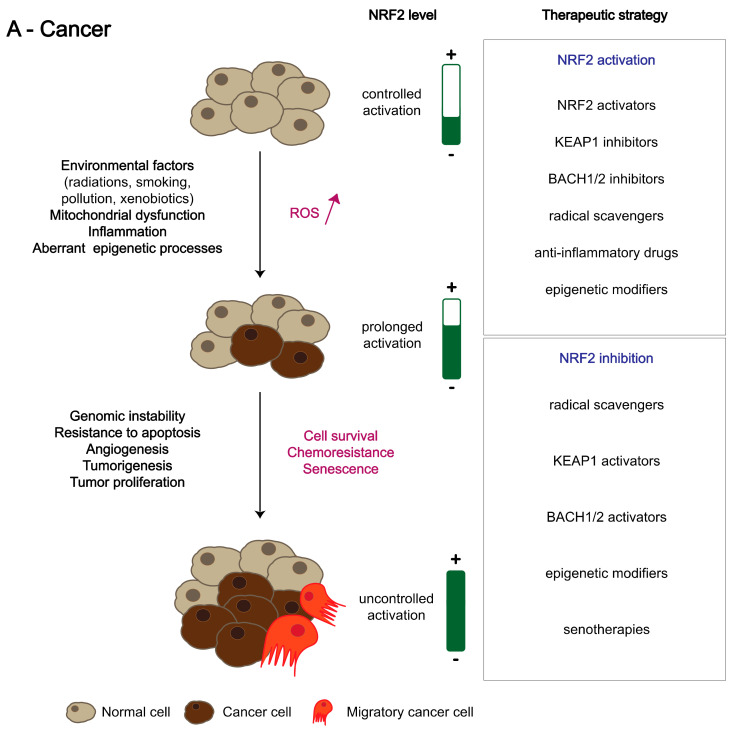

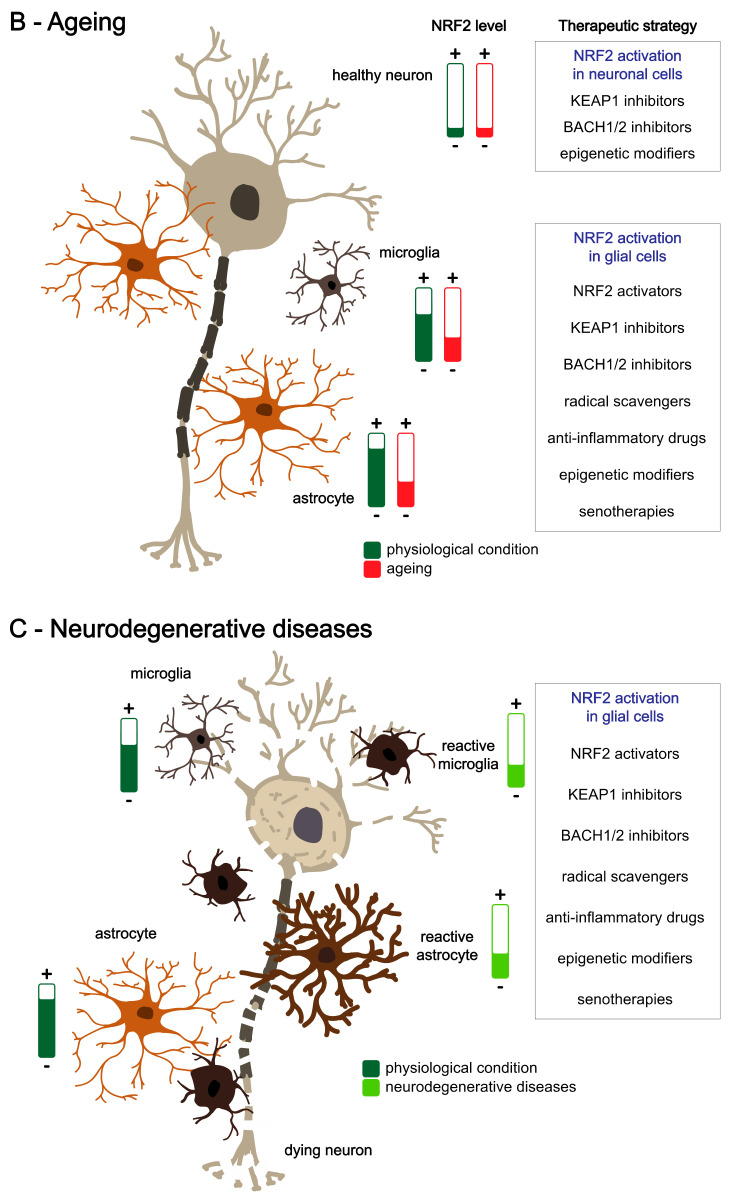
**NRF2-signalling-based therapeutic strategies in brain dysfunctions.** Targeting NRF2 pathway in brain diseases appears as a promising therapeutic strategy. Different options exist depending on the context (cancer (**A**), ageing (**B**), and neurodegenerative diseases (**C**)), NRF2 levels, and the cells to target (neuronal or glial cells). NRF2 and NRF2-negative regulators (KEAP1, BACH1, BACH2) can be targeted in these different conditions. The emergence of senotherapeutic drugs (senolytics, senomorphics) coupled to epigenetic modifiers (inhibitors/activators of histone acetyltransferases, histone deacetylases, histone demethylases, and histone methyltransferases) could provide a great number of translational opportunities and thus contribute to improving human health.

Targeting the NRF2 pathway in brain diseases appears to be context- but also time-dependent (Figure 4). Generally, NRF2 recruitment plays a protective role under physiological conditions, but it also promotes the development of many cancers once cancer is established [314] (Figure 4A). In early stages of cancer, NRF2 is able to thwart the deleterious effects of ROS, such as oxidative damages caused to cellular constituents (DNA, proteins, lipids). Pölönen and colleagues indeed described different NRF2 activation levels depending on the glioma WHO grades I-IV [245]. They reported that NRF2 activity was gradually higher in grades II to IV, whereas no constitutive NRF2 activity was detected in grade I. NRF2 expression may therefore be a useful biomarker to predict WHO grade and cellular behaviour of brain tumours [315]. In more advanced cancer stages, where NRF2 levels are higher, the therapeutic procedure would then be to decrease NRF2 activation in order to limit angiogenesis, cancer cell growth, and both chemo- and radio-resistance of cancer cells [316].

As life expectancy is increasing, the prevalence of neurological diseases is likely to rise accordingly. Age-dependent cognitive loss is observed in neurodegenerative disorders such as Alzheimer’s and Parkinson’s diseases [213]. In parallel, NRF2 activity has also been reported to decrease with age [182,184,215] (Figure 4B,C). As we previously mentioned, *NFE2L2* expression level is higher in glial cells (astrocytes, microglia) compared to neuronal cells in both mouse and human. Thus, discrepancies in targeting the NRF2 pathway could be brain-region- but also cell-type-dependent.

The future challenges will be to establish novel therapies to (a) specifically inhibit the NRF2 pathway in cancer cells, (b) increase NRF2 activation in specific cell types and/or brain regions, and (c) modulate NRF2 pathway in senescent cells. We believe that modulation of NRF2 signalling pathway by using specific food products and phytochemicals, dietary supplements, drugs, and epigenetic modifiers, alone or in combination, will help to limit inflammatory diseases, cancer progression, ageing process, and subsequently ageing-related diseases.

## Figures and Tables

**Table 1:** Methods for NRF2 activity monitoring.

Activities	Description	Methods	Principle	Observations	Refs
**Interaction KEAP1-NRF2**	The complex NRF2-KEAP1 is sequestered in the cytosol before being rapidly ubiquitinated and degradated by the proteasome.	Fluorescence Lifetime Imaging–Förster Resonance Energy Transfer (FLIM-FRET) approach in cells expressing fluorescently tagged NRF2 and KEAP1	evaluation of conformation changes in KEAP1-NRF2 complex in cells/establishment of NRF2 activation kinetics	Conformational changes (spatial distribution) can result in functional inactivation of the complex and consequential induction of an NRF2-mediated stress response.	[84,85,86]
**NRF2 translocation**	Free NRF2 will enter in the nucleus and activate the transcription of NRF2 target genes.	Western blots	evaluation of the NRF2 localisation (cytoplasmic versus nuclear levels)	increase in NRF2 level in the nucleus	[87,88,89]
		immunofluorescence (cells, tissues)	NRF2 staining in the nucleus	increase in NRF2 level in the nucleus	[87,90]
		transcriptomic analyses	analyses of NRF2 target genes expression	increase in gene expression	[53,87,91]
**NRF2 binding to AREs**	Free NRF2 enters in the nucleus and binds to specific sequences (Antioxidant Response Elements, ARE). This binding will activate the transcription of genes that have ARE sequences on their promoter.	ARE luciferase reporter kit	Cells are transfected by an ARE luciferase reporter vector constitutively expressing Renilla luciferase vector.	Amount of light generated after treatments is positively correlated with the ARE luciferase reporter activity as a consequence of NRF2 binding to ARE sequence.	[92]
		Electrophoretic-Mobility Shift Assay (EMSA)	Nuclear extracts are incubated with an NRF2 probe (oligonucleotide containing the ARE consensus sequence). Protein/DNA complexes are separated on a nondenaturing polyacrylamide gel and transferred to a membrane. Detection is performed using Streptavidin-HRP Conjugate and a chemiluminescent substrate.	The shifted bands corresponding to the protein/DNA complexes (interaction of NRF2 with its ARE-probe) are visualized.	[93]
		DNA-binding ELISA for activated NRF2 transcription factor	NRF2 transcription factor (from nuclear extracts) binds to DNA sequence (containing the NRF2 consensus binding site) immobilized in the well. Incubation with primary and secondary antibodies specifically quantifies the amount of activated transcription factor.	This method is 100-fold more sensitive than EMSAs. Colorimetric readout enables easy, quantitative analysis by spectrophotometry at 450 nm.	[94]
		NRF2/ARE luciferase reporter stable cell lines	After treatments, the NRF2 transcription factor will enter in the nucleus and bind to its response element. As a consequence, luciferase is expressed, and light will be generated in an enzymatic assay (addition of luciferin).	Amount of light generated is positively correlated with the level of NRF2 activation.	[95]
		high-content imaging of cell lines expressing fluorescent-tagged NRF2 (fluorescent protein reporter cell lines, expressing GFP tagged *Nfe2l2*)	High-throughput live confocal imaging is used to measure the temporal dynamics of the NRF2 pathway after treatments.	Fluorescent protein signal intensity is correlated to NRF2 response.	[96]
**GSH production**	NRF2 regulates GSH biosynthesizing enzymes (GCLM, GCLC) and plays a key role in the regulation of cellular GSH homeostasis	GSH quantification (GSH assay kit)	GSH is oxidized by the sulfhydryl reagent 5,5’-dithio-bis(2-nitrobenzoic acid). The formed derivative is measurable at 412 nm. The glutathione disulphide (GSSG) formed can be recycled to GSH by glutathione reductase in the presence of NADPH.	Increase in the GSH levels may confirm NRF2 recruitment. Glutathione quantification can be performed in any biological fluids, tissues, and cell extracts.	[97]
***Nfe2l2*** **manipulation**	activation/inhibition of the NRF2 signalling pathway and inactive-luciferase-tagged *Nfe2l2*, can be set up in transgenic mouse models	transgenic mouse models	Transgenic NRF2−/− mice (NRF2 pathway is downregulated) or KEAP1−/− mice (NRF2 pathway is upregulated) can be useful in order to verify the implication of the NRF2 pathway during pathologies or ageing.	Transgenic animal models are commercially available.	[98,99]
		OKD48 transgenic mice	The OKD48 construct has a 3xARE promoter, human *Nfe2l2*, and Flag-tagged luciferase. Upon stress, OKD48 is transcriptionally induced by the 3xARE element, and luciferase activity (luminescence) is observed only in cells experiencing oxidative stress.	OKD48-luc model is a highly specific and sensitive system for screening NRF2 activity	[100,101]

## Data Availability

Data is contained within the article.

## References

[1] Di Meo S., Napolitano G., Venditti P. (2019). Physiological and Pathological Role of ROS: Benefits and Limitations of Antioxidant Treatment. Int. J. Mol. Sci..

[2] Poyton R.O., Ball K.A., Castello P.R. (2009). Mitochondrial generation of free radicals and hypoxic signaling. Trends Endocrinol. Metab..

[3] Brand M.D. (2010). The sites and topology of mitochondrial superoxide production. Exp. Gerontol..

[4] Snezhkina A.V., Kudryavtseva A.V., Kardymon O.L., Savvateeva M.V., Melnikova N.V., Krasnov G.S., Dmitriev A.A. (2019). ROS Generation and Antioxidant Defense Systems in Normal and Malignant Cells. Oxid. Med. Cell. Longev..

[5] Panday A., Sahoo M.K., Osorio D., Batra S. (2015). NADPH oxidases: An overview from structure to innate immunity-associated pathologies. Cell. Mol. Immunol..

[6] Ayala A., Muñoz M.F., Argüelles S. (2014). Lipid peroxidation: Production, metabolism, and signaling mechanisms of malondialdehyde and 4-hydroxy-2-nonenal. Oxid. Med. Cell. Longev..

[7] Yin H., Xu L., Porter N.A. (2011). Free radical lipid peroxidation: Mechanisms and analysis. Chem. Rev..

[8] Pacher P., Beckman J.S., Liaudet L. (2007). Nitric Oxide and Peroxynitrite in Health and Disease. Physiol. Rev..

[9] Carraro E., Schilirò T., Biorci F., Romanazzi V., Degan R., Buonocore D., Verri M., Dossena M., Bonetta S., Gilli G. (2018). Physical Activity, Lifestyle Factors and Oxidative Stress in Middle Age Healthy Subjects. Int. J. Environ. Res. Public. Health.

[10] Di Meo S., Reed T.T., Venditti P., Victor V.M. (2016). Role of ROS and RNS Sources in Physiological and Pathological Conditions. Oxid. Med. Cell. Longev..

[11] Al-Gubory K.H. (2014). Environmental pollutants and lifestyle factors induce oxidative stress and poor prenatal development. Reprod. Biomed. Online.

[12] Aseervatham G.S.B., Sivasudha T., Jeyadevi R., Arul Ananth D. (2013). Environmental factors and unhealthy lifestyle influence oxidative stress in humans—An overview. Environ. Sci. Pollut. Res..

[13] Knaus U.G. (2021). Oxidants in Physiological Processes. Handb. Exp. Pharmacol..

[14] Bienert G.P., Schjoerring J.K., Jahn T.P. (2006). Membrane transport of hydrogen peroxide. Biochim. Biophys. Acta Biomembr..

[15] Jamil M., Debbarh H., Aboulmaouahib S., Aniq Filali O., Mounaji K., Zarqaoui M., Saadani B., Louanjli N., Cadi R. (2020). Reactive oxygen species in reproduction: Harmful, essential or both?. Zygote.

[16] Dennery P.A. (2010). Oxidative stress in development: Nature or nurture?. Free Radic. Biol. Med..

[17] Oswald M.C.W., Garnham N., Sweeney S.T., Landgraf M. (2018). Regulation of neuronal development and function by ROS. FEBS Lett..

[18] Chaudhari P., Ye Z., Jang Y.-Y. (2014). Roles of reactive oxygen species in the fate of stem cells. Antioxid. Redox Signal..

[19] Morimoto H., Iwata K., Ogonuki N., Inoue K., Atsuo O., Kanatsu-Shinohara M., Morimoto T., Yabe-Nishimura C., Shinohara T. (2013). ROS are required for mouse spermatogonial stem cell self-renewal. Cell Stem Cell.

[20] Sart S., Song L., Li Y. (2015). Controlling Redox Status for Stem Cell Survival, Expansion, and Differentiation. Oxid. Med. Cell. Longev..

[21] Bassoy E.Y., Walch M., Martinvalet D. (2021). Reactive Oxygen Species: Do They Play a Role in Adaptive Immunity?. Front. Immunol..

[22] Redza-Dutordoir M., Averill-Bates D.A. (2016). Activation of apoptosis signalling pathways by reactive oxygen species. Biochim. Biophys. Acta Mol. Cell Res..

[23] Santos A.L., Sinha S., Lindner A.B. (2018). The Good, the Bad, and the Ugly of ROS: New Insights on Aging and Aging-Related Diseases from Eukaryotic and Prokaryotic Model Organisms. Oxid. Med. Cell. Longev..

[24] Dhoke N.R., Geesala R., Das A. (2018). Low Oxidative Stress-Mediated Proliferation Via JNK-FOXO3a-Catalase Signaling in Transplanted Adult Stem Cells Promotes Wound Tissue Regeneration. Antioxid. Redox Signal..

[25] Lassègue B., Griendling K.K. (2004). Reactive oxygen species in hypertension: An update. Am. J. Hypertens..

[26] Kröller-Schön S., Daiber A., Steven S., Oelze M., Frenis K., Kalinovic S., Heimann A., Schmidt F.P., Pinto A., Kvandova M. (2018). Crucial role for Nox2 and sleep deprivation in aircraft noise-induced vascular and cerebral oxidative stress, inflammation, and gene regulation. Eur. Heart J..

[27] Steven S., Frenis K., Kalinovic S., Kvandova M., Oelze M., Helmstädter J., Hahad O., Filippou K., Kus K., Trevisan C. (2020). Exacerbation of adverse cardiovascular effects of aircraft noise in an animal model of arterial hypertension. Redox Biol..

[28] Kong H., Chandel N.S. (2018). Regulation of redox balance in cancer and T cells. J. Biol. Chem..

[29] Circu M.L., Aw T.Y. (2010). Reactive oxygen species, cellular redox systems, and apoptosis. Free Radic. Biol. Med..

[30] Zhang R., Brennan M.-L., Shen Z., MacPherson J.C., Schmitt D., Molenda C.E., Hazen S.L. (2002). Myeloperoxidase functions as a major enzymatic catalyst for initiation of lipid peroxidation at sites of inflammation. J. Biol. Chem..

[31] Funk C.D. (2001). Prostaglandins and leukotrienes: Advances in eicosanoid biology. Science.

[32] Lands W.E., Marshall P.J., Kulmacz R.J. (1985). Hydroperoxide availability in the regulation of the arachidonate cascade. Adv. Prostaglandin. Thromboxane. Leukot. Res..

[33] Bruegel M., Ceglarek U., Thiery J. (2009). Eicosanoids: Essential mediators in health and disease/Eicosanoide: Bedeutende Faktoren in der Homöostase und ihre Bedeutung in der Pathogenese multipler Erkrankungen. Lab. Med..

[34] Yui K., Imataka G., Nakamura H., Ohara N., Naito Y. (2015). Eicosanoids Derived From Arachidonic Acid and Their Family Prostaglandins and Cyclooxygenase in Psychiatric Disorders. Curr. Neuropharmacol..

[35] Tassoni D., Kaur G., Weisinger R.S., Sinclair A.J. (2008). The role of eicosanoids in the brain. Asia Pac. J. Clin. Nutr..

[36] Wymann M.P., Schneiter R. (2008). Lipid signalling in disease. Nat. Rev. Mol. Cell Biol..

[37] Ighodaro O.M., Akinloye O.A. (2018). First line defence antioxidants-superoxide dismutase (SOD), catalase (CAT) and glutathione peroxidase (GPX): Their fundamental role in the entire antioxidant defence grid. Alex. J. Med..

[38] Birben E., Sahiner U.M., Sackesen C., Erzurum S., Kalayci O. (2012). Oxidative stress and antioxidant defense. World Allergy Organ. J..

[39] Liguori I., Russo G., Curcio F., Bulli G., Aran L., Della-Morte D., Gargiulo G., Testa G., Cacciatore F., Bonaduce D. (2018). Oxidative stress, aging, and diseases. Clin. Interv. Aging.

[40] Flatt T. (2012). A new definition of aging?. Front. Genet..

[41] Harman D. (1956). Aging: A theory based on free radical and radiation chemistry. J. Gerontol..

[42] Sanada F., Taniyama Y., Muratsu J., Otsu R., Shimizu H., Rakugi H., Morishita R. (2018). Source of Chronic Inflammation in Aging. Front. Cardiovasc. Med..

[43] Tan B.L., Norhaizan M.E., Liew W.-P.-P., Sulaiman Rahman H. (2018). Antioxidant and Oxidative Stress: A Mutual Interplay in Age-Related Diseases. Front. Pharmacol..

[44] Chung H.Y., Kim D.H., Lee E.K., Chung K.W., Chung S., Lee B., Seo A.Y., Chung J.H., Jung Y.S., Im E. (2019). Redefining Chronic Inflammation in Aging and Age-Related Diseases: Proposal of the Senoinflammation Concept. Aging Dis..

[45] Ferrucci L., Fabbri E. (2018). Inflammageing: Chronic inflammation in ageing, cardiovascular disease, and frailty. Nat. Rev. Cardiol..

[46] Papaconstantinou J., Wang C.Z., Zhang M., Yang S., Deford J., Bulavin D.V., Ansari N.H. (2015). Attenuation of p38α MAPK stress response signaling delays the in vivo aging of skeletal muscle myofibers and progenitor cells. Aging.

[47] Akbari S., Amiri F.T., Naderi M., Shaki F., Seyedabadi M. (2022). Sodium arsenite accelerates D-galactose-induced aging in the testis of the rat: Evidence for mitochondrial oxidative damage, NF-kB, JNK, and apoptosis pathways. Toxicology.

[48] Ng A., Tam W.W., Zhang M.W., Ho C.S., Husain S.F., McIntyre R.S., Ho R.C. (2018). IL-1β, IL-6, TNF- α and CRP in Elderly Patients with Depression or Alzheimer’s disease: Systematic Review and Meta-Analysis. Sci. Rep..

[49] Chung H.Y., Sung B., Jung K.J., Zou Y., Yu B.P. (2006). The molecular inflammatory process in aging. Antioxid. Redox Signal..

[50] Zhu Y., Carvey P.M., Ling Z. (2006). Age-related changes in glutathione and glutathione-related enzymes in rat brain. Brain Res..

[51] Maher P. (2005). The effects of stress and aging on glutathione metabolism. Ageing Res. Rev..

[52] Bellezza I., Giambanco I., Minelli A., Donato R. (2018). Nrf2-Keap1 signaling in oxidative and reductive stress. Biochim. Biophys. Acta Mol. Cell Res..

[53] Tonelli C., Chio I.I.C., Tuveson D.A. (2018). Transcriptional Regulation by Nrf2. Antioxid. Redox Signal..

[54] Zhang X., Guo J., Wei X., Niu C., Jia M., Li Q., Meng D. (2018). Bach1: Function, Regulation, and Involvement in Disease. Oxid. Med. Cell. Longev..

[55] Davudian S., Mansoori B., Shajari N., Mohammadi A., Baradaran B. (2016). BACH1, the master regulator gene: A novel candidate target for cancer therapy. Gene.

[56] Dhakshinamoorthy S., Jain A.K., Bloom D.A., Jaiswal A.K. (2005). Bach1 Competes with Nrf2 Leading to Negative Regulation of the Antioxidant Response Element (ARE)-mediated NAD(P)H:Quinone Oxidoreductase 1 Gene Expression and Induction in Response to Antioxidants *. J. Biol. Chem..

[57] Yang L., Chen S., Zhao Q., Sun Y., Nie H. (2019). The Critical Role of Bach2 in Shaping the Balance between CD4+ T Cell Subsets in Immune-Mediated Diseases. Mediators Inflamm..

[58] Tsukumo S., Unno M., Muto A., Takeuchi A., Kometani K., Kurosaki T., Igarashi K., Saito T. (2013). Bach2 maintains T cells in a naive state by suppressing effector memory-related genes. Proc. Natl. Acad. Sci. USA.

[59] Hoshino H., Igarashi K. (2002). Expression of the oxidative stress-regulated transcription factor bach2 in differentiating neuronal cells. J. Biochem..

[60] He X., Chen M.G., Lin G.X., Ma Q. (2006). Arsenic induces NAD(P)H-quinone oxidoreductase I by disrupting the Nrf2 x Keap1 x Cul3 complex and recruiting Nrf2 x Maf to the antioxidant response element enhancer. J. Biol. Chem..

[61] He X., Ma Q. (2010). Critical cysteine residues of Kelch-like ECH-associated protein 1 in arsenic sensing and suppression of nuclear factor erythroid 2-related factor 2. J. Pharmacol. Exp. Ther..

[62] Yamamoto T., Suzuki T., Kobayashi A., Wakabayashi J., Maher J., Motohashi H., Yamamoto M. (2008). Physiological significance of reactive cysteine residues of Keap1 in determining Nrf2 activity. Mol. Cell. Biol..

[63] Zhang D.D., Hannink M. (2003). Distinct cysteine residues in Keap1 are required for Keap1-dependent ubiquitination of Nrf2 and for stabilization of Nrf2 by chemopreventive agents and oxidative stress. Mol. Cell. Biol..

[64] Boo Y.C. (2020). Natural Nrf2 Modulators for Skin Protection. Antioxidants.

[65] Osama A., Zhang J., Yao J., Yao X., Fang J. (2020). Nrf2: A dark horse in Alzheimer’s disease treatment. Ageing Res. Rev..

[66] Yang S.-Y., Pyo M.C., Nam M.-H., Lee K.-W. (2019). ERK/Nrf2 pathway activation by caffeic acid in HepG2 cells alleviates its hepatocellular damage caused by t-butylhydroperoxide-induced oxidative stress. BMC Complement. Altern. Med..

[67] Hu Y.-R., Ma H., Zou Z.-Y., He K., Xiao Y.-B., Wang Y., Feng M., Ye X.-L., Li X.-G. (2017). Activation of Akt and JNK/Nrf2/NQO1 pathway contributes to the protective effect of coptisine against AAPH-induced oxidative stress. Biomed. Pharmacother..

[68] Reddy N.M., Potteti H.R., Vegiraju S., Chen H.-J., Tamatam C.M., Reddy S.P. (2015). PI3K-AKT Signaling via Nrf2 Protects against Hyperoxia-Induced Acute Lung Injury, but Promotes Inflammation Post-Injury Independent of Nrf2 in Mice. PLoS ONE.

[69] Lee O.-H., Jain A.K., Papusha V., Jaiswal A.K. (2007). An auto-regulatory loop between stress sensors INrf2 and Nrf2 controls their cellular abundance. J. Biol. Chem..

[70] Culbreth M., Aschner M. (2018). GSK-3β, a double-edged sword in Nrf2 regulation: Implications for neurological dysfunction and disease. F1000Res.

[71] Chen X., Liu Y., Zhu J., Lei S., Dong Y., Li L., Jiang B., Tan L., Wu J., Yu S. (2016). GSK-3β downregulates Nrf2 in cultured cortical neurons and in a rat model of cerebral ischemia-reperfusion. Sci. Rep..

[72] Huo L., Li C.-W., Huang T.-H., Lam Y.C., Xia W., Tu C., Chang W.-C., Hsu J.L., Lee D.-F., Nie L. (2014). Activation of Keap1/Nrf2 signaling pathway by nuclear epidermal growth factor receptor in cancer cells. Am. J. Transl. Res..

[73] Tebay L.E., Robertson H., Durant S.T., Vitale S.R., Penning T.M., Dinkova-Kostova A.T., Hayes J.D. (2015). Mechanisms of activation of the transcription factor Nrf2 by redox stressors, nutrient cues, and energy status and the pathways through which it attenuates degenerative disease. Free Radic. Biol. Med..

[74] Fu J., Xiong Z., Huang C., Li J., Yang W., Han Y., Paiboonrungruan C., Major M.B., Chen K.-N., Kang X. (2019). Hyperactivity of the transcription factor Nrf2 causes metabolic reprogramming in mouse esophagus. J. Biol. Chem..

[75] Zhao J., Lin X., Meng D., Zeng L., Zhuang R., Huang S., Lv W., Hu J. (2020). Nrf2 Mediates Metabolic Reprogramming in Non-Small Cell Lung Cancer. Front. Oncol..

[76] Kasai S., Mimura J., Ozaki T., Itoh K. (2018). Emerging Regulatory Role of Nrf2 in Iron, Heme, and Hemoglobin Metabolism in Physiology and Disease. Front. Vet. Sci..

[77] Digaleh H., Kiaei M., Khodagholi F. (2013). Nrf2 and Nrf1 signaling and ER stress crosstalk: Implication for proteasomal degradation and autophagy. Cell. Mol. Life Sci..

[78] Bendavit G., Aboulkassim T., Hilmi K., Shah S., Batist G. (2016). Nrf2 Transcription Factor Can Directly Regulate mTOR: Linking Cytoprotective Gene Expression To A Major Metabolic Regulator That Generates Redox Activity. J. Biol. Chem..

[79] Lau A., Wang X.-J., Zhao F., Villeneuve N.F., Wu T., Jiang T., Sun Z., White E., Zhang D.D. (2010). A noncanonical mechanism of Nrf2 activation by autophagy deficiency: Direct interaction between Keap1 and p62. Mol. Cell. Biol..

[80] Jiang T., Harder B., Rojo de la Vega M., Wong P.K., Chapman E., Zhang D.D. (2015). p62 links autophagy and Nrf2 signaling. Free Radic. Biol. Med..

[81] Jain A., Lamark T., Sjøttem E., Larsen K.B., Awuh J.A., Øvervatn A., McMahon M., Hayes J.D., Johansen T. (2010). p62/SQSTM1 is a target gene for transcription factor NRF2 and creates a positive feedback loop by inducing antioxidant response element-driven gene transcription. J. Biol. Chem..

[82] Park J.S., Oh S.Y., Lee D.H., Lee Y.S., Sung S.H., Ji H.W., Lee M.J., Lee Y.-H., Rhee S.G., Bae S.H. (2016). p62/SQSTM1 is required for the protection against endoplasmic reticulum stress-induced apoptotic cell death. Free Radic. Res..

[83] Bian S., Zhao Y., Li F., Lu S., Yang S., Liu M., Wang S., Zhao D., Zhang W., Wang J. (2021). Knockdown of p62/sequestosome enhances ginsenoside Rh2-induced apoptosis in cervical cancer HeLa cells with no effect on autophagy. Biosci. Biotechnol. Biochem..

[84] Dikovskaya D., Dinkova-Kostova A.T. (2020). Measuring Changes in Keap1-Nrf2 Protein Complex Conformation in Individual Cells by FLIM-FRET. Curr. Protoc. Toxicol..

[85] Dikovskaya D., Appleton P.L., Bento-Pereira C., Dinkova-Kostova A.T. (2019). Measuring the Interaction of Transcription Factor Nrf2 with Its Negative Regulator Keap1 in Single Live Cells by an Improved FRET/FLIM Analysis. Chem. Res. Toxicol..

[86] Baird L., Swift S., Llères D., Dinkova-Kostova A.T. (2014). Monitoring Keap1-Nrf2 interactions in single live cells. Biotechnol. Adv..

[87] Heurtaux T., Kirchmeyer M., Koncina E., Felten P., Richart L., Uriarte Huarte O., Schohn H., Mittelbronn M. (2021). Apomorphine Reduces A53T α-Synuclein-Induced Microglial Reactivity Through Activation of NRF2 Signalling Pathway. Cell. Mol. Neurobiol..

[88] Zheng Y., Li L., Chen B., Fang Y., Lin W., Zhang T., Feng X., Tao X., Wu Y., Fu X. (2022). Chlorogenic acid exerts neuroprotective effect against hypoxia-ischemia brain injury in neonatal rats by activating Sirt1 to regulate the Nrf2-NF-κB signaling pathway. Cell Commun. Signal..

[89] Zhou L., Zhou M., Tan H., Xiao M. (2020). Cypermethrin-induced cortical neurons apoptosis via the Nrf2/ARE signaling pathway. Pestic. Biochem. Physiol..

[90] Zhang S., Jin S., Zhang S., Li Y.-Y., Wang H., Chen Y., Lu H. (2022). Vitexin protects against high glucose-induced endothelial cell apoptosis and oxidative stress via Wnt/β-catenin and Nrf2 signalling pathway. Arch. Physiol. Biochem..

[91] Mutter F.E., Park B.K., Copple I.M. (2015). Value of monitoring Nrf2 activity for the detection of chemical and oxidative stress. Biochem. Soc. Trans..

[92] Smirnova N.A., Haskew-Layton R.E., Basso M., Hushpulian D.M., Payappilly J.B., Speer R.E., Ahn Y.-H., Rakhman I., Cole P.A., Pinto J.T. (2011). Development of Neh2-luciferase reporter and its application for high throughput screening and real-time monitoring of Nrf2 activators. Chem. Biol..

[93] Wang Z., Chen G., Zhu W.-W., Zhou D. (2010). Activation of nuclear factor-erythroid 2-related factor 2 (Nrf2) in the basilar artery after subarachnoid hemorrhage in rats. Ann. Clin. Lab. Sci..

[94] Zhong Q., Mishra M., Kowluru R.A. (2013). Transcription factor Nrf2-mediated antioxidant defense system in the development of diabetic retinopathy. Investig. Ophthalmol. Vis. Sci..

[95] Wang X.J., Hayes J.D., Wolf C.R. (2006). Generation of a Stable Antioxidant Response Element–Driven Reporter Gene Cell Line and Its Use to Show Redox-Dependent Activation of Nrf2 by Cancer Chemotherapeutic Agents. Cancer Res..

[96] Bischoff L.J.M., Kuijper I.A., Schimming J.P., Wolters L., Braak B., Langenberg J.P., Noort D., Beltman J.B., van de Water B. (2019). A systematic analysis of Nrf2 pathway activation dynamics during repeated xenobiotic exposure. Arch. Toxicol..

[97] Rahman I., Kode A., Biswas S.K. (2006). Assay for quantitative determination of glutathione and glutathione disulfide levels using enzymatic recycling method. Nat. Protoc..

[98] Wang J., Zhu Q., Wang Y., Peng J., Shao L., Li X. (2022). Irisin protects against sepsis-associated encephalopathy by suppressing ferroptosis via activation of the Nrf2/GPX4 signal axis. Free Radic. Biol. Med..

[99] Hu Y., Wang P., Han K. (2022). Hydrogen Attenuated Inflammation Response and Oxidative in Hypoxic Ischemic Encephalopathy via Nrf2 Mediated the Inhibition of NLRP3 and NF-κB. Neuroscience.

[100] Oikawa D., Akai R., Tokuda M., Iwawaki T. (2012). A transgenic mouse model for monitoring oxidative stress. Sci. Rep..

[101] Inoue Y., Uchiyama A., Sekiguchi A., Yamazaki S., Fujiwara C., Yokoyama Y., Ogino S., Torii R., Hosoi M., Akai R. (2020). Protective effect of dimethyl fumarate for the development of pressure ulcers after cutaneous ischemia-reperfusion injury. Wound Repair Regen..

[102] Bazinet R.P., Layé S. (2014). Polyunsaturated fatty acids and their metabolites in brain function and disease. Nat. Rev. Neurosci..

[103] Cobley J.N., Fiorello M.L., Bailey D.M. (2018). 13 reasons why the brain is susceptible to oxidative stress. Redox Biol..

[104] Ren X., Zou L., Zhang X., Branco V., Wang J., Carvalho C., Holmgren A., Lu J. (2017). Redox Signaling Mediated by Thioredoxin and Glutathione Systems in the Central Nervous System. Antioxid. Redox Signal..

[105] Lee K.H., Cha M., Lee B.H. (2021). Crosstalk between Neuron and Glial Cells in Oxidative Injury and Neuroprotection. Int. J. Mol. Sci..

[106] Dringen R., Gutterer J.M., Hirrlinger J. (2000). Glutathione metabolism in brain: Metabolic interaction between astrocytes and neurons in the defense against reactive oxygen species. Eur. J. Biochem..

[107] Chen Y., Qin C., Huang J., Tang X., Liu C., Huang K., Xu J., Guo G., Tong A., Zhou L. (2020). The role of astrocytes in oxidative stress of central nervous system: A mixed blessing. Cell Prolif..

[108] Simpson D.S.A., Oliver P.L. (2020). ROS Generation in Microglia: Understanding Oxidative Stress and Inflammation in Neurodegenerative Disease. Antioxidants.

[109] Liddell J.R. (2017). Are Astrocytes the Predominant Cell Type for Activation of Nrf2 in Aging and Neurodegeneration?. Antioxidants.

[110] He F., Ru X., Wen T. (2020). NRF2, a Transcription Factor for Stress Response and Beyond. Int. J. Mol. Sci..

[111] Boas S.M., Joyce K.L., Cowell R.M. (2021). The NRF2-Dependent Transcriptional Regulation of Antioxidant Defense Pathways: Relevance for Cell Type-Specific Vulnerability to Neurodegeneration and Therapeutic Intervention. Antioxidants.

[112] Asanuma M., Miyazaki I. (2021). Glutathione and Related Molecules in Parkinsonism. Int. J. Mol. Sci..

[113] Burton N.C., Kensler T.W., Guilarte T.R. (2006). In vivo modulation of the Parkinsonian phenotype by Nrf2. Neurotoxicology.

[114] Hubbs A.F., Benkovic S.A., Miller D.B., O’Callaghan J.P., Battelli L., Schwegler-Berry D., Ma Q. (2007). Vacuolar leukoencephalopathy with widespread astrogliosis in mice lacking transcription factor Nrf2. Am. J. Pathol..

[115] Jakel R.J., Townsend J.A., Kraft A.D., Johnson J.A. (2007). Nrf2-mediated protection against 6-hydroxydopamine. Brain Res..

[116] Innamorato N.G., Jazwa A., Rojo A.I., García C., Fernández-Ruiz J., Grochot-Przeczek A., Stachurska A., Jozkowicz A., Dulak J., Cuadrado A. (2010). Different susceptibility to the Parkinson’s toxin MPTP in mice lacking the redox master regulator Nrf2 or its target gene heme oxygenase-1. PLoS ONE.

[117] Boyanapalli S.S.S., Paredes-Gonzalez X., Fuentes F., Zhang C., Guo Y., Pung D., Saw C.L.L., Kong A.-N.T. (2014). Nrf2 knockout attenuates the anti-inflammatory effects of phenethyl isothiocyanate and curcumin. Chem. Res. Toxicol..

[118] Johnson D.A., Amirahmadi S., Ward C., Fabry Z., Johnson J.A. (2010). The absence of the pro-antioxidant transcription factor Nrf2 exacerbates experimental autoimmune encephalomyelitis. Toxicol. Sci..

[119] Rojo A.I., Pajares M., García-Yagüe A.J., Buendia I., Van Leuven F., Yamamoto M., López M.G., Cuadrado A. (2018). Deficiency in the transcription factor NRF2 worsens inflammatory parameters in a mouse model with combined tauopathy and amyloidopathy. Redox Biol..

[120] Branca C., Ferreira E., Nguyen T.-V., Doyle K., Caccamo A., Oddo S. (2017). Genetic reduction of Nrf2 exacerbates cognitive deficits in a mouse model of Alzheimer’s disease. Hum. Mol. Genet..

[121] Zhu J., Wang H., Sun Q., Ji X., Zhu L., Cong Z., Zhou Y., Liu H., Zhou M. (2013). Nrf2 is required to maintain the self-renewal of glioma stem cells. BMC Cancer.

[122] Tarantini S., Valcarcel-Ares M.N., Yabluchanskiy A., Tucsek Z., Hertelendy P., Kiss T., Gautam T., Zhang X.A., Sonntag W.E., de Cabo R. (2018). Nrf2 Deficiency Exacerbates Obesity-Induced Oxidative Stress, Neurovascular Dysfunction, Blood-Brain Barrier Disruption, Neuroinflammation, Amyloidogenic Gene Expression, and Cognitive Decline in Mice, Mimicking the Aging Phenotype. J. Gerontol. A Biol. Sci. Med. Sci..

[123] Sandberg M., Patil J., D’Angelo B., Weber S.G., Mallard C. (2014). NRF2-regulation in brain health and disease: Implication of cerebral inflammation. Neuropharmacology.

[124] Deshmukh P., Unni S., Krishnappa G., Padmanabhan B. (2017). The Keap1–Nrf2 pathway: Promising therapeutic target to counteract ROS-mediated damage in cancers and neurodegenerative diseases. Biophys. Rev..

[125] Brandes M.S., Gray N.E. (2020). NRF2 as a Therapeutic Target in Neurodegenerative Diseases. ASN Neuro.

[126] Anandhan A., Nguyen N., Syal A., Dreher L.A., Dodson M., Zhang D.D., Madhavan L. (2021). NRF2 Loss Accentuates Parkinsonian Pathology and Behavioral Dysfunction in Human α-Synuclein Overexpressing Mice. Aging Dis..

[127] Bono S., Feligioni M., Corbo M. (2021). Impaired antioxidant KEAP1-NRF2 system in amyotrophic lateral sclerosis: NRF2 activation as a potential therapeutic strategy. Mol. Neurodegener..

[128] Cuadrado A. (2022). Brain-Protective Mechanisms of Transcription Factor NRF2: Toward a Common Strategy for Neurodegenerative Diseases. Annu. Rev. Pharmacol. Toxicol..

[129] Furman D., Campisi J., Verdin E., Carrera-Bastos P., Targ S., Franceschi C., Ferrucci L., Gilroy D.W., Fasano A., Miller G.W. (2019). Chronic inflammation in the etiology of disease across the life span. Nat. Med..

[130] Liu T., Zhang L., Joo D., Sun S.-C. (2017). NF-κB signaling in inflammation. Signal Transduct. Target. Ther..

[131] Christian F., Smith E.L., Carmody R.J. (2016). The Regulation of NF-κB Subunits by Phosphorylation. Cells.

[132] Wardyn J.D., Ponsford A.H., Sanderson C.M. (2015). Dissecting molecular cross-talk between Nrf2 and NF-κB response pathways. Biochem. Soc. Trans..

[133] Sivandzade F., Prasad S., Bhalerao A., Cucullo L. (2019). NRF2 and NF-κB interplay in cerebrovascular and neurodegenerative disorders: Molecular mechanisms and possible therapeutic approaches. Redox Biol..

[134] Ganesh Yerra V., Negi G., Sharma S.S., Kumar A. (2013). Potential therapeutic effects of the simultaneous targeting of the Nrf2 and NF-κB pathways in diabetic neuropathy. Redox Biol..

[135] Li W., Khor T.O., Xu C., Shen G., Jeong W.-S., Yu S., Kong A.-N. (2008). Activation of Nrf2-antioxidant signaling attenuates NFkappaB-inflammatory response and elicits apoptosis. Biochem. Pharmacol..

[136] Ahmed S.M.U., Luo L., Namani A., Wang X.J., Tang X. (2017). Nrf2 signaling pathway: Pivotal roles in inflammation. Biochim. Biophys. Acta Mol. Basis Dis..

[137] Saha S., Buttari B., Panieri E., Profumo E., Saso L. (2020). An Overview of Nrf2 Signaling Pathway and Its Role in Inflammation. Molecules.

[138] Pae H.-O., Chung H.-T. (2009). Heme oxygenase-1: Its therapeutic roles in inflammatory diseases. Immune Netw..

[139] Drummond G.S., Baum J., Greenberg M., Lewis D., Abraham N.G. (2019). HO-1 overexpression and underexpression: Clinical implications. Arch. Biochem. Biophys..

[140] Subedi L., Lee J.H., Yumnam S., Ji E., Kim S.Y. (2019). Anti-Inflammatory Effect of Sulforaphane on LPS-Activated Microglia Potentially through JNK/AP-1/NF-κB Inhibition and Nrf2/HO-1 Activation. Cells.

[141] Nitti M., Furfaro A.L., Mann G.E. (2020). Heme Oxygenase Dependent Bilirubin Generation in Vascular Cells: A Role in Preventing Endothelial Dysfunction in Local Tissue Microenvironment?. Front. Physiol..

[142] Helou D.G., Martin S.F., Pallardy M., Chollet-Martin S., Kerdine-Römer S. (2019). Nrf2 Involvement in Chemical-Induced Skin Innate Immunity. Front. Immunol..

[143] Krajka-Kuźniak V., Baer-Dubowska W. (2021). Modulation of Nrf2 and NF-κB Signaling Pathways by Naturally Occurring Compounds in Relation to Cancer Prevention and Therapy. Are Combinations Better Than Single Compounds?. Int. J. Mol. Sci..

[144] Yu M., Li H., Liu Q., Liu F., Tang L., Li C., Yuan Y., Zhan Y., Xu W., Li W. (2011). Nuclear factor p65 interacts with Keap1 to repress the Nrf2-ARE pathway. Cell. Signal..

[145] Hu B., Wei H., Song Y., Chen M., Fan Z., Qiu R., Zhu W., Xu W., Wang F. (2020). NF-κB and Keap1 Interaction Represses Nrf2-Mediated Antioxidant Response in Rabbit Hemorrhagic Disease Virus Infection. J. Virol..

[146] Bellezza I., Mierla A.L., Minelli A. (2010). Nrf2 and NF-κB and Their Concerted Modulation in Cancer Pathogenesis and Progression. Cancers.

[147] Guo X., Hong S., He H., Zeng Y., Chen Y., Mo X., Li J., Li L., Steinmetz R., Liu Q. (2020). NFκB promotes oxidative stress-induced necrosis and ischemia/reperfusion injury by inhibiting Nrf2-ARE pathway. Free Radic. Biol. Med..

[148] Gao W., Guo L., Yang Y., Wang Y., Xia S., Gong H., Zhang B.-K., Yan M. (2021). Dissecting the Crosstalk Between Nrf2 and NF-κB Response Pathways in Drug-Induced Toxicity. Front. Cell Dev. Biol..

[149] Liu G.-H., Qu J., Shen X. (2008). NF-kappaB/p65 antagonizes Nrf2-ARE pathway by depriving CBP from Nrf2 and facilitating recruitment of HDAC3 to MafK. Biochim. Biophys. Acta.

[150] Jin W., Zhu L., Guan Q., Chen G., Wang Q.F., Yin H.X., Hang C.H., Shi J.X., Wang H.D. (2008). Influence of Nrf2 genotype on pulmonary NF-kappaB activity and inflammatory response after traumatic brain injury. Ann. Clin. Lab. Sci..

[151] Pan H., Wang H., Wang X., Zhu L., Mao L. (2012). The absence of Nrf2 enhances NF-κB-dependent inflammation following scratch injury in mouse primary cultured astrocytes. Mediators Inflamm..

[152] Wang K., Zheng M., Lester K.L., Han Z. (2019). Light-induced Nrf2-/- mice as atrophic age-related macular degeneration model and treatment with nanoceria laden injectable hydrogel. Sci. Rep..

[153] Chang S.-H., Lee J.-S., Yun U.J., Park K.W. (2021). A Role of Stress Sensor Nrf2 in Stimulating Thermogenesis and Energy Expenditure. Biomedicines.

[154] Jardim N.S., Müller S.G., Pase F.M., Nogueira C.W. (2021). Nuclear Factor [Erythroid-derived 2]-like 2 and Mitochondrial Transcription Factor A Contribute to Moderate-intensity Swimming Effectiveness against Memory Impairment in Young Mice Induced by Concomitant Exposure to a High-calorie Diet during the Early Life Period. Neuroscience.

[155] Stevenson R.J., Francis H.M. (2017). The hippocampus and the regulation of human food intake. Psychol. Bull..

[156] Mattson M.P. (2012). Energy intake and exercise as determinants of brain health and vulnerability to injury and disease. Cell Metab..

[157] Vasconcelos A.R., Dos Santos N.B., Scavone C., Munhoz C.D. (2019). Nrf2/ARE Pathway Modulation by Dietary Energy Regulation in Neurological Disorders. Front. Pharmacol..

[158] Matsui S., Sasaki T., Kohno D., Yaku K., Inutsuka A., Yokota-Hashimoto H., Kikuchi O., Suga T., Kobayashi M., Yamanaka A. (2018). Neuronal SIRT1 regulates macronutrient-based diet selection through FGF21 and oxytocin signalling in mice. Nat. Commun..

[159] Gälman C., Lundåsen T., Kharitonenkov A., Bina H.A., Eriksson M., Hafström I., Dahlin M., Amark P., Angelin B., Rudling M. (2008). The circulating metabolic regulator FGF21 is induced by prolonged fasting and PPARalpha activation in man. Cell Metab..

[160] Laeger T., Henagan T.M., Albarado D.C., Redman L.M., Bray G.A., Noland R.C., Münzberg H., Hutson S.M., Gettys T.W., Schwartz M.W. (2014). FGF21 is an endocrine signal of protein restriction. J. Clin. Investig..

[161] Solon-Biet S.M., Cogger V.C., Pulpitel T., Heblinski M., Wahl D., McMahon A.C., Warren A., Durrant-Whyte J., Walters K.A., Krycer J.R. (2016). Defining the Nutritional and Metabolic Context of FGF21 Using the Geometric Framework. Cell Metab..

[162] Lundsgaard A.-M., Fritzen A.M., Sjøberg K.A., Myrmel L.S., Madsen L., Wojtaszewski J.F.P., Richter E.A., Kiens B. (2017). Circulating FGF21 in humans is potently induced by short term overfeeding of carbohydrates. Mol. Metab..

[163] Jensen-Cody S.O., Flippo K.H., Claflin K.E., Yavuz Y., Sapouckey S.A., Walters G.C., Usachev Y.M., Atasoy D., Gillum M.P., Potthoff M.J. (2020). FGF21 Signals to Glutamatergic Neurons in the Ventromedial Hypothalamus to Suppress Carbohydrate Intake. Cell Metab..

[164] Sajja R.K., Prasad S., Tang S., Kaisar M.A., Cucullo L. (2017). Blood-brain barrier disruption in diabetic mice is linked to Nrf2 signaling deficits: Role of ABCB10?. Neurosci. Lett..

[165] Yu Z., Lin L., Jiang Y., Chin I., Wang X., Li X., Lo E.H., Wang X. (2019). Recombinant FGF21 Protects Against Blood-Brain Barrier Leakage Through Nrf2 Upregulation in Type 2 Diabetes Mice. Mol. Neurobiol..

[166] Furusawa Y., Uruno A., Yagishita Y., Higashi C., Yamamoto M. (2014). Nrf2 induces fibroblast growth factor 21 in diabetic mice. Genes Cells.

[167] Sáenz de Urturi D., Buqué X., Porteiro B., Folgueira C., Mora A., Delgado T.C., Prieto-Fernández E., Olaizola P., Gómez-Santos B., Apodaka-Biguri M. (2022). Methionine adenosyltransferase 1a antisense oligonucleotides activate the liver-brown adipose tissue axis preventing obesity and associated hepatosteatosis. Nat. Commun..

[168] Yagishita Y., Uruno A., Fukutomi T., Saito R., Saigusa D., Pi J., Fukamizu A., Sugiyama F., Takahashi S., Yamamoto M. (2017). Nrf2 Improves Leptin and Insulin Resistance Provoked by Hypothalamic Oxidative Stress. Cell Rep..

[169] Bösl M.R., Seldin M.F., Nishimura S., Taketo M. (1995). Cloning, structural analysis and mapping of the mouse selenocysteine tRNA([Ser]Sec) gene (Trsp). Mol. Gen. Genet..

[170] Shen G., Zhou L., Liu W., Cui Y., Xie W., Chen H., Yu W., Li W., Li H. (2017). Di(2-ethylhexyl)phthalate Alters the Synthesis and β-Oxidation of Fatty Acids and Hinders ATP Supply in Mouse Testes via UPLC-Q-Exactive Orbitrap MS-Based Metabonomics Study. J. Agric. Food Chem..

[171] Wang Y., Li L., Wang Y., Zhu X., Jiang M., Song E., Song Y. (2018). New application of the commercial sweetener rebaudioside a as a hepatoprotective candidate: Induction of the Nrf2 signaling pathway. Eur. J. Pharmacol..

[172] Xia S.-F., Shao J., Zhao S.-Y., Qiu Y.-Y., Teng L.-P., Huang W., Wang S.-S., Cheng X.-R., Jiang Y.-Y. (2018). Niga-ichigoside F1 ameliorates high-fat diet-induced hepatic steatosis in male mice by Nrf2 activation. Food Funct..

[173] Niu Y., He J., Ahmad H., Shen M., Zhao Y., Gan Z., Zhang L., Zhong X., Wang C., Wang T. (2019). Dietary Curcumin Supplementation Increases Antioxidant Capacity, Upregulates Nrf2 and Hmox1 Levels in the Liver of Piglet Model with Intrauterine Growth Retardation. Nutrients.

[174] Amos D., Cook C., Santanam N. (2019). Omega 3 rich diet modulates energy metabolism via GPR120-Nrf2 crosstalk in a novel antioxidant mouse model. Biochim. Biophys. Acta Mol. Cell Biol. Lipids.

[175] Rajendran P., Ammar R.B., Al-Saeedi F.J., Mohamed M.E., ElNaggar M.A., Al-Ramadan S.Y., Bekhet G.M., Soliman A.M. (2020). Kaempferol Inhibits Zearalenone-Induced Oxidative Stress and Apoptosis via the PI3K/Akt-Mediated Nrf2 Signaling Pathway: In Vitro and In Vivo Studies. Int. J. Mol. Sci..

[176] Erten F., Orhan C., Tuzcu M., Er B., Defo Deeh P.B., Sahin N., Özercan I.H., Juturu V., Sahin K. (2020). Salacia chinensis exerts its antidiabetic effect by modulating glucose-regulated proteins and transcription factors in high-fat diet fed-streptozotocin-induced type 2 diabetic rats. J. Food Biochem..

[177] Illesca P., Valenzuela R., Espinosa A., Echeverría F., Soto-Alarcon S., Campos C., Rodriguez A., Vargas R., Magrone T., Videla L.A. (2020). Protective Effects of Eicosapentaenoic Acid Plus Hydroxytyrosol Supplementation Against White Adipose Tissue Abnormalities in Mice Fed a High-Fat Diet. Molecules.

[178] Abrescia P., Treppiccione L., Rossi M., Bergamo P. (2020). Modulatory role of dietary polyunsaturated fatty acids in Nrf2-mediated redox homeostasis. Prog. Lipid Res..

[179] Mohamed A.B., Rémond D., Gual-Grau A., Bernalier-Donnadille A., Capel F., Michalski M.-C., Laugerette F., Cohade B., Hafnaoui N., Béchet D. (2021). A Mix of Dietary Fibres Changes Interorgan Nutrients Exchanges and Muscle-Adipose Energy Handling in Overfed Mini-Pigs. Nutrients.

[180] Bruns D.R., Drake J.C., Biela L.M., Peelor F.F., Miller B.F., Hamilton K.L. (2015). Nrf2 Signaling and the Slowed Aging Phenotype: Evidence from Long-Lived Models. Oxid. Med. Cell. Longev..

[181] Dodson M., Anandhan A., Zhang D.D., Madhavan L. (2021). An NRF2 Perspective on Stem Cells and Ageing. Front. Aging.

[182] Matsumaru D., Motohashi H. (2021). The KEAP1-NRF2 System in Healthy Aging and Longevity. Antioxidants.

[183] Lewis K.N., Wason E., Edrey Y.H., Kristan D.M., Nevo E., Buffenstein R. (2015). Regulation of Nrf2 signaling and longevity in naturally long-lived rodents. Proc. Natl. Acad. Sci. USA.

[184] Zhang H., Davies K.J.A., Forman H.J. (2015). Oxidative stress response and Nrf2 signaling in aging. Free Radic. Biol. Med..

[185] Silva-Palacios A., Ostolga-Chavarría M., Zazueta C., Königsberg M. (2018). Nrf2: Molecular and epigenetic regulation during aging. Ageing Res. Rev..

[186] Tsay H.J., Wang P., Wang S.L., Ku H.H. (2000). Age-associated changes of superoxide dismutase and catalase activities in the rat brain. J. Biomed. Sci..

[187] Samarghandian S., Azimi-Nezhad M., Samini F. (2015). Preventive effect of safranal against oxidative damage in aged male rat brain. Exp. Anim..

[188] Berr C., Balansard B., Arnaud J., Roussel A.M., Alpérovitch A. (2000). Cognitive decline is associated with systemic oxidative stress: The EVA study. Etude du Vieillissement Artériel. J. Am. Geriatr. Soc..

[189] Baierle M., Nascimento S.N., Moro A.M., Brucker N., Freitas F., Gauer B., Durgante J., Bordignon S., Zibetti M., Trentini C.M. (2015). Relationship between inflammation and oxidative stress and cognitive decline in the institutionalized elderly. Oxid. Med. Cell. Longev..

[190] Schöttker B., Saum K.-U., Jansen E.H.J.M., Holleczek B., Brenner H. (2016). Associations of metabolic, inflammatory and oxidative stress markers with total morbidity and multi-morbidity in a large cohort of older German adults. Age Ageing.

[191] Hajjar I., Hayek S.S., Goldstein F.C., Martin G., Jones D.P., Quyyumi A. (2018). Oxidative stress predicts cognitive decline with aging in healthy adults: An observational study. J. Neuroinflamm..

[192] Cuadrado A., Manda G., Hassan A., Alcaraz M.J., Barbas C., Daiber A., Ghezzi P., León R., López M.G., Oliva B. (2018). Transcription Factor NRF2 as a Therapeutic Target for Chronic Diseases: A Systems Medicine Approach. Pharmacol. Rev..

[193] Hushpulian D.M., Ammal Kaidery N., Ahuja M., Poloznikov A.A., Sharma S.M., Gazaryan I.G., Thomas B. (2021). Challenges and Limitations of Targeting the Keap1-Nrf2 Pathway for Neurotherapeutics: Bach1 De-Repression to the Rescue. Front. Aging Neurosci..

[194] Han K., Jin X., Guo X., Cao G., Tian S., Song Y., Zuo Y., Yu P., Gao G., Chang Y.-Z. (2021). Nrf2 knockout altered brain iron deposition and mitigated age-related motor dysfunction in aging mice. Free Radic. Biol. Med..

[195] Liu Z., Han K., Huo X., Yan B., Gao M., Lv X., Yu P., Gao G., Chang Y.-Z. (2020). Nrf2 knockout dysregulates iron metabolism and increases the hemolysis through ROS in aging mice. Life Sci..

[196] Bao W.-D., Pang P., Zhou X.-T., Hu F., Xiong W., Chen K., Wang J., Wang F., Xie D., Hu Y.-Z. (2021). Loss of ferroportin induces memory impairment by promoting ferroptosis in Alzheimer’s disease. Cell Death Differ..

[197] Raha A.A., Biswas A., Henderson J., Chakraborty S., Holland A., Friedland R.P., Mukaetova-Ladinska E., Zaman S., Raha-Chowdhury R. (2022). Interplay of Ferritin Accumulation and Ferroportin Loss in Ageing Brain: Implication for Protein Aggregation in Down Syndrome Dementia, Alzheimer’s, and Parkinson’s Diseases. Int. J. Mol. Sci..

[198] Kubben N., Zhang W., Wang L., Voss T.C., Yang J., Qu J., Liu G.-H., Misteli T. (2016). Repression of the Antioxidant NRF2 Pathway in Premature Aging. Cell.

[199] Sadowska-Bartosz I., Bartosz G. (2014). Effect of antioxidants supplementation on aging and longevity. BioMed Res. Int..

[200] Conti V., Izzo V., Corbi G., Russomanno G., Manzo V., De Lise F., Di Donato A., Filippelli A. (2016). Antioxidant Supplementation in the Treatment of Aging-Associated Diseases. Front. Pharmacol..

[201] Grilc N.K., Sova M., Kristl J. (2021). Drug Delivery Strategies for Curcumin and Other Natural Nrf2 Modulators of Oxidative Stress-Related Diseases. Pharmaceutics.

[202] Malavolta M., Bracci M., Santarelli L., Sayeed M.A., Pierpaoli E., Giacconi R., Costarelli L., Piacenza F., Basso A., Cardelli M. (2018). Inducers of Senescence, Toxic Compounds, and Senolytics: The Multiple Faces of Nrf2-Activating Phytochemicals in Cancer Adjuvant Therapy. Mediators Inflamm..

[203] Kulkarni A.S., Gubbi S., Barzilai N. (2020). Benefits of Metformin in Attenuating the Hallmarks of Aging. Cell Metab..

[204] Ngoi N.Y.L., Liew A.Q.X., Chong S.J.F., Davids M.S., Clement M.-V., Pervaiz S. (2021). The redox-senescence axis and its therapeutic targeting. Redox Biol..

[205] Zhang L., Pitcher L.E., Prahalad V., Niedernhofer L.J., Robbins P.D. (2022). Targeting cellular senescence with senotherapeutics: Senolytics and senomorphics. FEBS J..

[206] Nayeri Rad A., Shams G., Avelar R.A., Morowvat M.H., Ghasemi Y. (2022). Potential senotherapeutic candidates and their combinations derived from transcriptional connectivity and network measures. Inform. Med. Unlocked.

[207] Sikora E., Bielak-Zmijewska A., Dudkowska M., Krzystyniak A., Mosieniak G., Wesierska M., Wlodarczyk J. (2021). Cellular Senescence in Brain Aging. Front. Aging Neurosci..

[208] Martínez-Cué C., Rueda N. (2020). Cellular Senescence in Neurodegenerative Diseases. Front. Cell. Neurosci..

[209] Ogrodnik M., Evans S.A., Fielder E., Victorelli S., Kruger P., Salmonowicz H., Weigand B.M., Patel A.D., Pirtskhalava T., Inman C.L. (2021). Whole-body senescent cell clearance alleviates age-related brain inflammation and cognitive impairment in mice. Aging Cell.

[210] Krzystyniak A., Wesierska M., Petrazzo G., Gadecka A., Dudkowska M., Bielak-Zmijewska A., Mosieniak G., Figiel I., Wlodarczyk J., Sikora E. (2022). Combination of dasatinib and quercetin improves cognitive abilities in aged male Wistar rats, alleviates inflammation and changes hippocampal synaptic plasticity and histone H3 methylation profile. Aging.

[211] Yuan H., Xu Y., Luo Y., Wang N.-X., Xiao J.-H. (2021). Role of Nrf2 in cell senescence regulation. Mol. Cell. Biochem..

[212] Saha S., Buttari B., Profumo E., Tucci P., Saso L. (2022). A Perspective on Nrf2 Signaling Pathway for Neuroinflammation: A Potential Therapeutic Target in Alzheimer’s and Parkinson’s Diseases. Front. Cell. Neurosci..

[213] Uddin M.S., Stachowiak A., Mamun A.A., Tzvetkov N.T., Takeda S., Atanasov A.G., Bergantin L.B., Abdel-Daim M.M., Stankiewicz A.M. (2018). Autophagy and Alzheimer’s Disease: From Molecular Mechanisms to Therapeutic Implications. Front. Aging Neurosci..

[214] Dinkova-Kostova A.T., Kostov R.V., Kazantsev A.G. (2018). The role of Nrf2 signaling in counteracting neurodegenerative diseases. FEBS J..

[215] Schmidlin C.J., Dodson M.B., Madhavan L., Zhang D.D. (2019). Redox Regulation by NRF2 in Aging and Disease. Free Radic. Biol. Med..

[216] Joshi G., Gan K.A., Johnson D.A., Johnson J.A. (2015). Increased Alzheimer’s disease-like pathology in the APP/ PS1ΔE9 mouse model lacking Nrf2 through modulation of autophagy. Neurobiol. Aging.

[217] Ren P., Chen J., Li B., Zhang M., Yang B., Guo X., Chen Z., Cheng H., Wang P., Wang S. (2020). Nrf2 Ablation Promotes Alzheimer’s Disease-Like Pathology in APP/PS1 Transgenic Mice: The Role of Neuroinflammation and Oxidative Stress. Oxid. Med. Cell. Longev..

[218] Kanninen K., Heikkinen R., Malm T., Rolova T., Kuhmonen S., Leinonen H., Ylä-Herttuala S., Tanila H., Levonen A.-L., Koistinaho M. (2009). Intrahippocampal injection of a lentiviral vector expressing Nrf2 improves spatial learning in a mouse model of Alzheimer’s disease. Proc. Natl. Acad. Sci. USA.

[219] Jiwaji Z., Tiwari S.S., Avilés-Reyes R.X., Hooley M., Hampton D., Torvell M., Johnson D.A., McQueen J., Baxter P., Sabari-Sankar K. (2022). Reactive astrocytes acquire neuroprotective as well as deleterious signatures in response to Tau and Aß pathology. Nat. Commun..

[220] Ramsey C.P., Glass C.A., Montgomery M.B., Lindl K.A., Ritson G.P., Chia L.A., Hamilton R.L., Chu C.T., Jordan-Sciutto K.L. (2007). Expression of Nrf2 in neurodegenerative diseases. J. Neuropathol. Exp. Neurol..

[221] Delaidelli A., Richner M., Jiang L., van der Laan A., Bergholdt Jul Christiansen I., Ferreira N., Nyengaard J.R., Vægter C.B., Jensen P.H., Mackenzie I.R. (2021). α-Synuclein pathology in Parkinson disease activates homeostatic NRF2 anti-oxidant response. Acta Neuropathol. Commun..

[222] Barone M.C., Sykiotis G.P., Bohmann D. (2011). Genetic activation of Nrf2 signaling is sufficient to ameliorate neurodegenerative phenotypes in a Drosophila model of Parkinson’s disease. Dis. Model. Mech..

[223] Zhang C., Zhao M., Wang B., Su Z., Guo B., Qin L., Zhang W., Zheng R. (2021). The Nrf2-NLRP3-caspase-1 axis mediates the neuroprotective effects of Celastrol in Parkinson’s disease. Redox Biol..

[224] Zhong Y., Cai X., Ding L., Liao J., Liu X., Huang Y., Chen X., Long L. (2022). Nrf2 Inhibits the Progression of Parkinson’s Disease by Upregulating AABR07032261.5 to Repress Pyroptosis. J. Inflamm. Res..

[225] Chen J., Rusnak M., Lombroso P.J., Sidhu A. (2009). Dopamine promotes striatal neuronal apoptotic death via ERK signaling cascades. Eur. J. Neurosci..

[226] Rojo A.I., Innamorato N.G., Martín-Moreno A.M., De Ceballos M.L., Yamamoto M., Cuadrado A. (2010). Nrf2 regulates microglial dynamics and neuroinflammation in experimental Parkinson’s disease. Glia.

[227] Lastres-Becker I., Ulusoy A., Innamorato N.G., Sahin G., Rábano A., Kirik D., Cuadrado A. (2012). α-Synuclein expression and Nrf2 deficiency cooperate to aggravate protein aggregation, neuronal death and inflammation in early-stage Parkinson’s disease. Hum. Mol. Genet..

[228] Gan L., Vargas M.R., Johnson D.A., Johnson J.A. (2012). Astrocyte-specific overexpression of Nrf2 delays motor pathology and synuclein aggregation throughout the CNS in the alpha-synuclein mutant (A53T) mouse model. J. Neurosci..

[229] Vargas M.R., Burton N.C., Kutzke J., Gan L., Johnson D.A., Schäfer M., Werner S., Johnson J.A. (2013). Absence of Nrf2 or its selective overexpression in neurons and muscle does not affect survival in ALS-linked mutant hSOD1 mouse models. PLoS ONE.

[230] Sigfridsson E., Marangoni M., Johnson J.A., Hardingham G.E., Fowler J.H., Horsburgh K. (2018). Astrocyte-specific overexpression of Nrf2 protects against optic tract damage and behavioural alterations in a mouse model of cerebral hypoperfusion. Sci. Rep..

[231] Daniels C.M.L., Austin E.V., Rockney D.E., Jacka E.M., Hagemann T.L., Johnson D.A., Johnson J.A., Messing A. (2012). Beneficial Effects of Nrf2 Overexpression in a Mouse Model of Alexander Disease. J. Neurosci..

[232] Jenner P. (2003). Oxidative stress in Parkinson’s disease. Ann. Neurol..

[233] Gan L., Johnson J.A. (2014). Oxidative damage and the Nrf2-ARE pathway in neurodegenerative diseases. Biochim. Biophys. Acta.

[234] Vinish M., Anand A., Prabhakar S. (2011). Altered oxidative stress levels in Indian Parkinson’s disease patients with PARK2 mutations. Acta Biochim. Pol..

[235] Manoharan S., Guillemin G.J., Abiramasundari R.S., Essa M.M., Akbar M., Akbar M.D. (2016). The Role of Reactive Oxygen Species in the Pathogenesis of Alzheimer’s Disease, Parkinson’s Disease, and Huntington’s Disease: A Mini Review. Oxid. Med. Cell. Longev..

[236] Wei Z., Li X., Li X., Liu Q., Cheng Y. (2018). Oxidative Stress in Parkinson’s Disease: A Systematic Review and Meta-Analysis. Front. Mol. Neurosci..

[237] Pan H., Wang H., Zhu L., Wang X., Cong Z., Sun K., Fan Y. (2013). The involvement of Nrf2-ARE pathway in regulation of apoptosis in human glioblastoma cell U251. Neurol. Res..

[238] Zhou Y., Wang H.-D., Zhu L., Cong Z.-X., Li N., Ji X.-J., Pan H., Wang J.-W., Li W.-C. (2013). Knockdown of Nrf2 enhances autophagy induced by temozolomide in U251 human glioma cell line. Oncol. Rep..

[239] Cong Z.-X., Wang H.-D., Wang J.-W., Zhou Y., Pan H., Zhang D.-D., Zhu L. (2013). ERK and PI3K signaling cascades induce Nrf2 activation and regulate cell viability partly through Nrf2 in human glioblastoma cells. Oncol. Rep..

[240] Fan Z., Wirth A.-K., Chen D., Wruck C.J., Rauh M., Buchfelder M., Savaskan N. (2017). Nrf2-Keap1 pathway promotes cell proliferation and diminishes ferroptosis. Oncogenesis.

[241] Ji X.-J., Chen S.-H., Zhu L., Pan H., Zhou Y., Li W., You W.-C., Gao C.-C., Zhu J.-H., Jiang K. (2013). Knockdown of NF-E2-related factor 2 inhibits the proliferation and growth of U251MG human glioma cells in a mouse xenograft model. Oncol. Rep..

[242] Ji X., Wang H., Zhu J., Zhu L., Pan H., Li W., Zhou Y., Cong Z., Yan F., Chen S. (2014). Knockdown of Nrf2 suppresses glioblastoma angiogenesis by inhibiting hypoxia-induced activation of HIF-1α. Int. J. Cancer.

[243] Haapasalo J., Nordfors K., Granberg K.J., Kivioja T., Nykter M., Haapasalo H., Soini Y. (2018). NRF2, DJ1 and SNRX1 and their prognostic impact in astrocytic gliomas. Histol. Histopathol..

[244] Ji X., Wang H., Zhu J., Tang Y., Zhou Y., Zhu L., Gao C., Li W., You W., Yu B. (2013). Correlation of Nrf2 and HIF-1α in glioblastoma and their relationships to clinicopathologic features and survival. Neurol. Res..

[245] Pölönen P., Jawahar Deen A., Leinonen H.M., Jyrkkänen H.-K., Kuosmanen S., Mononen M., Jain A., Tuomainen T., Pasonen-Seppänen S., Hartikainen J.M. (2019). Nrf2 and SQSTM1/p62 jointly contribute to mesenchymal transition and invasion in glioblastoma. Oncogene.

[246] Kanamori M., Higa T., Sonoda Y., Murakami S., Dodo M., Kitamura H., Taguchi K., Shibata T., Watanabe M., Suzuki H. (2015). Activation of the NRF2 pathway and its impact on the prognosis of anaplastic glioma patients. Neuro-Oncology.

[247] Li K., Ouyang L., He M., Luo M., Cai W., Tu Y., Pi R., Liu A. (2017). IDH1 R132H mutation regulates glioma chemosensitivity through Nrf2 pathway. Oncotarget.

[248] Ahmad F., Dixit D., Sharma V., Kumar A., Joshi S.D., Sarkar C., Sen E. (2016). Nrf2-driven TERT regulates pentose phosphate pathway in glioblastoma. Cell Death Dis..

[249] Tsakonas G., Martín-Bernabé A., Rounis K., Moreno-Ruiz P., Botling J., De Petris L., Ylipää A., Mezheyeuski A., Micke P., Östman A. (2021). High Density of NRF2 Expression in Malignant Cells Is Associated with Increased Risk of CNS Metastasis in Early-Stage NSCLC. Cancers.

[250] Aljohani H.M., Aittaleb M., Furgason J.M., Amaya P., Deeb A., Chalmers J.J., Bahassi E.M. (2018). Genetic mutations associated with lung cancer metastasis to the brain. Mutagenesis.

[251] Cong Z.-X., Wang H.-D., Zhou Y., Wang J.-W., Pan H., Zhang D.-D., Zhang L., Zhu L. (2014). Temozolomide and irradiation combined treatment-induced Nrf2 activation increases chemoradiation sensitivity in human glioblastoma cells. J. Neurooncol..

[252] Rocha C.R.R., Reily Rocha A., Molina Silva M., Rodrigues Gomes L., Teatin Latancia M., Andrade Tomaz M., de Souza I., Karolynne Seregni Monteiro L., Menck C.F.M. (2020). Revealing Temozolomide Resistance Mechanisms via Genome-Wide CRISPR Libraries. Cells.

[253] Escoll M., Lastra D., Robledinos-Antón N., Wandosell F., Antón I.M., Cuadrado A. (2020). WIP Modulates Oxidative Stress through NRF2/KEAP1 in Glioblastoma Cells. Antioxidants.

[254] Beltzig L., Schwarzenbach C., Leukel P., Frauenknecht K.B.M., Sommer C., Tancredi A., Hegi M.E., Christmann M., Kaina B. (2022). Senescence Is the Main Trait Induced by Temozolomide in Glioblastoma Cells. Cancers.

[255] Yagishita Y., Gatbonton-Schwager T.N., McCallum M.L., Kensler T.W. (2020). Current Landscape of NRF2 Biomarkers in Clinical Trials. Antioxidants.

[256] Kornberg R.D., Lorch Y. (1999). Twenty-five years of the nucleosome, fundamental particle of the eukaryote chromosome. Cell.

[257] Baker M. (2011). Making sense of chromatin states. Nat. Methods.

[258] Ohnmacht J., May P., Sinkkonen L., Krüger R. (2020). Missing heritability in Parkinson’s disease: The emerging role of non-coding genetic variation. J. Neural Transm..

[259] Blackwood E.M., Kadonaga J.T. (1998). Going the distance: A current view of enhancer action. Science.

[260] Krivega I., Dean A. (2012). Enhancer and promoter interactions-long distance calls. Curr. Opin. Genet. Dev..

[261] Correa F., Mallard C., Nilsson M., Sandberg M. (2011). Activated microglia decrease histone acetylation and Nrf2-inducible anti-oxidant defence in astrocytes: Restoring effects of inhibitors of HDACs, p38 MAPK and GSK3β. Neurobiol. Dis..

[262] Wang B., Zhu X., Kim Y., Li J., Huang S., Saleem S., Li R., Xu Y., Dore S., Cao W. (2012). Histone deacetylase inhibition activates transcription factor Nrf2 and protects against cerebral ischemic damage. Free Radic. Biol. Med..

[263] Li Z., Xu L., Tang N., Xu Y., Ye X., Shen S., Niu X., Lu S., Chen Z. (2014). The polycomb group protein EZH2 inhibits lung cancer cell growth by repressing the transcription factor Nrf2. FEBS Lett..

[264] Chorley B.N., Campbell M.R., Wang X., Karaca M., Sambandan D., Bangura F., Xue P., Pi J., Kleeberger S.R., Bell D.A. (2012). Identification of novel NRF2-regulated genes by ChIP-Seq: Influence on retinoid X receptor alpha. Nucleic Acids Res..

[265] Malhotra D., Portales-Casamar E., Singh A., Srivastava S., Arenillas D., Happel C., Shyr C., Wakabayashi N., Kensler T.W., Wasserman W.W. (2010). Global mapping of binding sites for Nrf2 identifies novel targets in cell survival response through ChIP-Seq profiling and network analysis. Nucleic Acids Res..

[266] He C.H., Gong P., Hu B., Stewart D., Choi M.E., Choi A.M., Alam J. (2001). Identification of activating transcription factor 4 (ATF4) as an Nrf2-interacting protein. Implication for heme oxygenase-1 gene regulation. J. Biol. Chem..

[267] Zhang J., Ohta T., Maruyama A., Hosoya T., Nishikawa K., Maher J.M., Shibahara S., Itoh K., Yamamoto M. (2006). BRG1 interacts with Nrf2 to selectively mediate HO-1 induction in response to oxidative stress. Mol. Cell. Biol..

[268] Alam J., Killeen E., Gong P., Naquin R., Hu B., Stewart D., Ingelfinger J.R., Nath K.A. (2003). Heme activates the heme oxygenase-1 gene in renal epithelial cells by stabilizing Nrf2. Am. J. Physiol. Renal Physiol..

[269] Sekine H., Okazaki K., Ota N., Shima H., Katoh Y., Suzuki N., Igarashi K., Ito M., Motohashi H., Yamamoto M. (2016). The Mediator Subunit MED16 Transduces NRF2-Activating Signals into Antioxidant Gene Expression. Mol. Cell. Biol..

[270] Nioi P., Nguyen T., Sherratt P.J., Pickett C.B. (2005). The carboxy-terminal Neh3 domain of Nrf2 is required for transcriptional activation. Mol. Cell. Biol..

[271] Kim J.-H., Yu S., Chen J.D., Kong A.N. (2013). The nuclear cofactor RAC3/AIB1/SRC-3 enhances Nrf2 signaling by interacting with transactivation domains. Oncogene.

[272] Pan H., Guan D., Liu X., Li J., Wang L., Wu J., Zhou J., Zhang W., Ren R., Zhang W. (2016). SIRT6 safeguards human mesenchymal stem cells from oxidative stress by coactivating NRF2. Cell Res..

[273] Guttman M., Amit I., Garber M., French C., Lin M.F., Feldser D., Huarte M., Zuk O., Carey B.W., Cassady J.P. (2009). Chromatin signature reveals over a thousand highly conserved large non-coding RNAs in mammals. Nature.

[274] Ikeda Y., Sugawara A., Taniyama Y., Uruno A., Igarashi K., Arima S., Ito S., Takeuchi K. (2000). Suppression of rat thromboxane synthase gene transcription by peroxisome proliferator-activated receptor gamma in macrophages via an interaction with NRF2. J. Biol. Chem..

[275] Shah N.M., Rushworth S.A., Murray M.Y., Bowles K.M., MacEwan D.J. (2013). Understanding the role of NRF2-regulated miRNAs in human malignancies. Oncotarget.

[276] Joo M.S., Lee C.G., Koo J.H., Kim S.G. (2013). miR-125b transcriptionally increased by Nrf2 inhibits AhR repressor, which protects kidney from cisplatin-induced injury. Cell Death Dis..

[277] Kwak M.-K., Wakabayashi N., Itoh K., Motohashi H., Yamamoto M., Kensler T.W. (2003). Modulation of gene expression by cancer chemopreventive dithiolethiones through the Keap1-Nrf2 pathway. Identification of novel gene clusters for cell survival. J. Biol. Chem..

[278] Handy D.E., Castro R., Loscalzo J. (2011). Epigenetic modifications: Basic mechanisms and role in cardiovascular disease. Circulation.

[279] Marstrand T.T., Storey J.D. (2014). Identifying and mapping cell-type-specific chromatin programming of gene expression. Proc. Natl. Acad. Sci. USA.

[280] Winick-Ng W., Kukalev A., Harabula I., Zea-Redondo L., Szabó D., Meijer M., Serebreni L., Zhang Y., Bianco S., Chiariello A.M. (2021). Cell-type specialization is encoded by specific chromatin topologies. Nature.

[281] Czamara D., Eraslan G., Page C.M., Lahti J., Lahti-Pulkkinen M., Hämäläinen E., Kajantie E., Laivuori H., Villa P.M., Reynolds R.M. (2019). Integrated analysis of environmental and genetic influences on cord blood DNA methylation in new-borns. Nat. Commun..

[282] Breton C.V., Landon R., Kahn L.G., Enlow M.B., Peterson A.K., Bastain T., Braun J., Comstock S.S., Duarte C.S., Hipwell A. (2021). Exploring the evidence for epigenetic regulation of environmental influences on child health across generations. Commun. Biol..

[283] Wang Z., Pan Q., Gendron P., Zhu W., Guo F., Cen S., Wainberg M.A., Liang C. (2016). CRISPR/Cas9-Derived Mutations Both Inhibit HIV-1 Replication and Accelerate Viral Escape. Cell Rep..

[284] Levings D.C., Wang X., Kohlhase D., Bell D.A., Slattery M. (2018). A distinct class of antioxidant response elements is consistently activated in tumors with NRF2 mutations. Redox Biol..

[285] Oyake T., Itoh K., Motohashi H., Hayashi N., Hoshino H., Nishizawa M., Yamamoto M., Igarashi K. (1996). Bach proteins belong to a novel family of BTB-basic leucine zipper transcription factors that interact with MafK and regulate transcription through the NF-E2 site. Mol. Cell. Biol..

[286] Itoh-Nakadai A., Hikota R., Muto A., Kometani K., Watanabe-Matsui M., Sato Y., Kobayashi M., Nakamura A., Miura Y., Yano Y. (2014). The transcription repressors Bach2 and Bach1 promote B cell development by repressing the myeloid program. Nat. Immunol..

[287] Shin S., Wakabayashi N., Misra V., Biswal S., Lee G.H., Agoston E.S., Yamamoto M., Kensler T.W. (2007). NRF2 modulates aryl hydrocarbon receptor signaling: Influence on adipogenesis. Mol. Cell. Biol..

[288] Chan K., Lu R., Chang J.C., Kan Y.W. (1996). NRF2, a member of the NFE2 family of transcription factors, is not essential for murine erythropoiesis, growth, and development. Proc. Natl. Acad. Sci. USA.

[289] Rushworth S.A., Zaitseva L., Murray M.Y., Shah N.M., Bowles K.M., MacEwan D.J. (2012). The high Nrf2 expression in human acute myeloid leukemia is driven by NF-κB and underlies its chemo-resistance. Blood.

[290] Tao S., Wang S., Moghaddam S.J., Ooi A., Chapman E., Wong P.K., Zhang D.D. (2014). Oncogenic KRAS confers chemoresistance by upregulating NRF2. Cancer Res..

[291] Saunders A., Macosko E.Z., Wysoker A., Goldman M., Krienen F.M., de Rivera H., Bien E., Baum M., Bortolin L., Wang S. (2018). Molecular Diversity and Specializations among the Cells of the Adult Mouse Brain. Cell.

[292] Murphy T.H., Yu J., Ng R., Johnson D.A., Shen H., Honey C.R., Johnson J.A. (2001). Preferential expression of antioxidant response element mediated gene expression in astrocytes. J. Neurochem..

[293] Shih A.Y., Johnson D.A., Wong G., Kraft A.D., Jiang L., Erb H., Johnson J.A., Murphy T.H. (2003). Coordinate Regulation of Glutathione Biosynthesis and Release by Nrf2-Expressing Glia Potently Protects Neurons from Oxidative Stress. J. Neurosci..

[294] Kraft A.D., Johnson D.A., Johnson J.A. (2004). Nuclear Factor E2-Related Factor 2-Dependent Antioxidant Response Element Activation by tert-Butylhydroquinone and Sulforaphane Occurring Preferentially in Astrocytes Conditions Neurons against Oxidative Insult. J. Neurosci..

[295] Bell K.F.S., Al-Mubarak B., Martel M.-A., McKay S., Wheelan N., Hasel P., Márkus N.M., Baxter P., Deighton R.F., Serio A. (2015). Neuronal development is promoted by weakened intrinsic antioxidant defences due to epigenetic repression of Nrf2. Nat. Commun..

[296] Narasimhan M., Patel D., Vedpathak D., Rathinam M., Henderson G., Mahimainathan L. (2012). Identification of novel microRNAs in post-transcriptional control of Nrf2 expression and redox homeostasis in neuronal, SH-SY5Y cells. PLoS ONE.

[297] Jing X., Shi H., Zhang C., Ren M., Han M., Wei X., Zhang X., Lou H. (2015). Dimethyl fumarate attenuates 6-OHDA-induced neurotoxicity in SH-SY5Y cells and in animal model of Parkinson’s disease by enhancing Nrf2 activity. Neuroscience.

[298] Ahuja M., Ammal Kaidery N., Yang L., Calingasan N., Smirnova N., Gaisin A., Gaisina I.N., Gazaryan I., Hushpulian D.M., Kaddour-Djebbar I. (2016). Distinct Nrf2 Signaling Mechanisms of Fumaric Acid Esters and Their Role in Neuroprotection against 1-Methyl-4-Phenyl-1,2,3,6-Tetrahydropyridine-Induced Experimental Parkinson’s-Like Disease. J. Neurosci..

[299] Campolo M., Casili G., Biundo F., Crupi R., Cordaro M., Cuzzocrea S., Esposito E. (2017). The Neuroprotective Effect of Dimethyl Fumarate in an MPTP-Mouse Model of Parkinson’s Disease: Involvement of Reactive Oxygen Species/Nuclear Factor-κB/Nuclear Transcription Factor Related to NF-E2. Antioxid. Redox Signal..

[300] Campolo M., Casili G., Lanza M., Filippone A., Paterniti I., Cuzzocrea S., Esposito E. (2018). Multiple mechanisms of dimethyl fumarate in amyloid β-induced neurotoxicity in human neuronal cells. J. Cell. Mol. Med..

[301] Rajput M.S., Nirmal N.P., Rathore D., Dahima R. (2020). Dimethyl Fumarate Mitigates Tauopathy in Aβ-Induced Neuroblastoma SH-SY5Y Cells. Neurochem. Res..

[302] Sun X., Suo X., Xia X., Yu C., Dou Y. (2022). Dimethyl Fumarate is a Potential Therapeutic Option for Alzheimer’s Disease. J. Alzheimers Dis..

[303] Singh A., Venkannagari S., Oh K.H., Zhang Y.-Q., Rohde J.M., Liu L., Nimmagadda S., Sudini K., Brimacombe K.R., Gajghate S. (2016). Small Molecule Inhibitor of NRF2 Selectively Intervenes Therapeutic Resistance in KEAP1-Deficient NSCLC Tumors. ACS Chem. Biol..

[304] Okazaki K., Anzawa H., Liu Z., Ota N., Kitamura H., Onodera Y., Alam M.M., Matsumaru D., Suzuki T., Katsuoka F. (2020). Enhancer remodeling promotes tumor-initiating activity in NRF2-activated non-small cell lung cancers. Nat. Commun..

[305] Preissl S., Fang R., Huang H., Zhao Y., Raviram R., Gorkin D.U., Zhang Y., Sos B.C., Afzal V., Dickel D.E. (2018). Single-nucleus analysis of accessible chromatin in developing mouse forebrain reveals cell-type-specific transcriptional regulation. Nat. Neurosci..

[306] Mich J.K., Graybuck L.T., Hess E.E., Mahoney J.T., Kojima Y., Ding Y., Somasundaram S., Miller J.A., Kalmbach B.E., Radaelli C. (2021). Functional enhancer elements drive subclass-selective expression from mouse to primate neocortex. Cell Rep..

[307] Gui Y., Grzyb K., Thomas M.H., Ohnmacht J., Garcia P., Buttini M., Skupin A., Sauter T., Sinkkonen L. (2021). Single-nuclei chromatin profiling of ventral midbrain reveals cell identity transcription factors and cell-type-specific gene regulatory variation. Epigenetics Chromatin.

[308] Ahuja M., Ammal Kaidery N., Attucks O.C., McDade E., Hushpulian D.M., Gaisin A., Gaisina I., Ahn Y.H., Nikulin S., Poloznikov A. (2021). Bach1 derepression is neuroprotective in a mouse model of Parkinson’s disease. Proc. Natl. Acad. Sci. USA.

[309] Wang X.J., Hayes J.D., Henderson C.J., Wolf C.R. (2007). Identification of retinoic acid as an inhibitor of transcription factor Nrf2 through activation of retinoic acid receptor alpha. Proc. Natl. Acad. Sci. USA.

[310] Armand E.J., Li J., Xie F., Luo C., Mukamel E.A. (2021). Single-Cell Sequencing of Brain Cell Transcriptomes and Epigenomes. Neuron.

[311] Maurano M.T., Humbert R., Rynes E., Thurman R.E., Haugen E., Wang H., Reynolds A.P., Sandstrom R., Qu H., Brody J. (2012). Systematic localization of common disease-associated variation in regulatory DNA. Science.

[312] Schaub M.A., Boyle A.P., Kundaje A., Batzoglou S., Snyder M. (2012). Linking disease associations with regulatory information in the human genome. Genome Res..

[313] Deplancke B., Alpern D., Gardeux V. (2016). The Genetics of Transcription Factor DNA Binding Variation. Cell.

[314] Rojo de la Vega M., Chapman E., Zhang D.D. (2018). NRF2 and the Hallmarks of Cancer. Cancer Cell.

[315] Tsai W.-C., Hueng D.-Y., Lin C.-R., Yang T.C.K., Gao H.-W. (2016). Nrf2 Expressions Correlate with WHO Grades in Gliomas and Meningiomas. Int. J. Mol. Sci..

[316] Wu S., Lu H., Bai Y. (2019). Nrf2 in cancers: A double-edged sword. Cancer Med..

